# ATR and PKMYT1 Inhibition Resensitizes a Subset of TNBC Patient-Derived Models to Carboplatin, Inducing Mitotic Catastrophe

**DOI:** 10.1158/2767-9764.CRC-25-0044

**Published:** 2026-05-12

**Authors:** Juliet Guay, Hellen Kuasne, Catherine Chabot, Kathryn Bozek, Yasamin Majedi, Marguerite Buchanan, Adriana Aguilar-Mahecha, Eric Bareke, Benjamin Ulmer, Tim Kong, Kangning Yang, Minyan Liao, Oluwadara Elebute, Ruining Guo, Anie Monast, Geneviève Morin, Sidong Huang, Morag Park, Mark Basik

**Affiliations:** 1 Lady Davis Institute, McGill University, Montreal, Canada.; 2 Department of Experimental Medicine, McGill University, Montreal, Canada.; 3 Rosalind & Morris Goodman Cancer Research Institute, McGill University, Montreal, Canada.; 4 Department of Human Genetic, McGill University, Montreal, Canada.; 5 Department of Biochemistry, McGill University, Montreal, Canada.; 6 Department of Oncology, McGill University, Montreal, Canada.; 7 Department of Surgery, McGill University, Montreal, Canada.

## Abstract

Triple-negative breast cancer (TNBC) is associated with poor prognosis and is mainly treated with chemotherapy-based regimens, often including carboplatin. Resistance to carboplatin is a common clinical issue that is either initially present or develops with treatment. Overcoming this resistance is a significant clinical challenge, which highlights the need for novel therapeutic strategies. We used a pooled short hairpin RNA screening approach with a chemoresistant TNBC patient-derived xenograft (PDX) cell line (PDXC) to identify targets in which knockdown would enhance the efficacy of carboplatin. This screening led to the identification of the ataxia telangiectasia and Rad3-related (ATR) gene as a key therapeutic vulnerability. Inhibiting ATR with BAY1895344 or AZD6738 resensitized carboplatin-resistant PDXCs and PDXs to carboplatin, resulting in an increase in DNA damage and apoptosis. ATR inhibition prevents carboplatin-resistant cells from effectively engaging the S and G_2_–M checkpoints required for DNA repair, leading to mitotic catastrophe. We further identified that the addition of ATR inhibitors to carboplatin enabled the FOXM1-targeted gene program, leading to premature passage into mitosis. Moreover, targeting PKMYT1, a regulator of cyclin-dependent kinase 1 controlling the G_2_–M checkpoint, through knockdown or with the novel PKMYT1 inhibitor RP-6306, also enhanced carboplatin efficacy in our TNBC PDXC. Molecular factors associated with the response to the ATR inhibitor/carboplatin combination included low RNA levels of PKMYT1. These results underscore the pivotal roles of ATR and PKMYT1 in mediating resistance to carboplatin in TNBC and support targeting these pathways to overcome carboplatin resistance in this disease.

**Significance:**

Patients with TNBC are mostly treated with chemotherapy, including carboplatin. Given that patients often develop resistance to carboplatin, finding a way to resensitize them to this agent is paramount. Our work highlights the use of ATR and PKMYT1 inhibitors to resensitize chemoresistant patient-derived models to carboplatin. These studies in patient-derived models establish the basis for novel therapeutic drug combinations to overcome carboplatin resistance in patients with TNBC.

## Introduction

Triple-negative breast cancers (TNBC) do not express estrogen receptors (ER) or progesterone receptors, nor do they have overexpression or amplification of the human epidermal growth factor 2 receptor (*HER2*), and are associated with a poor prognosis (1–3). Despite recent advances in TNBC treatment that have led to the recent approvals of antibody–drug conjugates like sacituzumab govitecan, PARP inhibitors like olaparib and talazoparib, and immunotherapies like atezolizumab and pembrolizumab, TNBC treatment still relies heavily on chemotherapy, including in the neoadjuvant setting in early TNBC (4). A key component of chemotherapy in TNBC is carboplatin as it was shown to improve pathologic complete response in combination with anthracyclines and taxanes (5). Nonetheless, at least 35% of early TNBCs are resistant to carboplatin-containing neoadjuvant therapy, and patients with metastatic TNBC inevitably develop resistance to carboplatin. Uncovering ways to overcome carboplatin resistance in TNBC remains a major unmet need in this disease.

Carboplatin is a platinum-based chemotherapeutic drug, an alkylating agent that works primarily by binding to DNA, inducing DNA cross-links, thereby interfering with DNA synthesis and inducing replicative stress, ultimately leading to cell death. Several studies have uncovered different mechanisms involved in carboplatin resistance, including decreased drug uptake, increased drug detoxification, increased DNA repair processes, and repression of apoptotic signaling, among others (6). In TNBCs, carboplatin resistance has been associated with deregulation of the mitotic checkpoint (7). However, none of these mechanisms have led to successful therapeutic treatments to overcome carboplatin resistance in the clinic.

Deficiencies in DNA repair pathways, including homologous recombination repair, particularly due to loss-of-function mutations in the *BRCA1* and *BRCA2* genes, are linked to increased sensitivity of TNBCs to carboplatin (8). This suggests that the combination of carboplatin with targeted therapies that disrupt the DNA damage response (DDR) may be able to overcome resistance to carboplatin. Ataxia telangiectasia and Rad3-related (ATR) is a serine/threonine kinase that plays a pivotal role in the DDR and replicative stress. ATR regulates the cell cycle in response to DNA damage via its main effector CHK1 in S-phase and in G_2_–M (9, 10), leading to cell-cycle arrest and facilitating DNA repair. In the presence of stalled replication forks, ATR mediates the activation of the mitotic inhibitor WEE1 through CHK1 activation, which results in the inactivation of cyclin-dependent kinase 1 (CDK1), preventing entry into M-phase.

ATR inhibition can significantly sensitize cancer cells to DNA-damaging agents such as chemotherapy, including carboplatin and radiation, as shown in extensive preclinical and clinical studies (11, 12). Defects in DDR mechanisms, such as *ATM* mutations affecting homologous recombination, dysfunctional *TP53*, and elevated levels of replication stress, correlate with sensitivity to ATR inhibitors (13, 14). Several ATR inhibitors are in preclinical development and are being tested in phase I and phase II clinical trials as single agents or in combination with DNA-damaging agents or with immune checkpoint inhibitors (13). Notably, berzosertib (M6620/VX-970/VE-822; ref. 15) and ceralasertib (AZD6738; ref. 16) have been tested in combination with platinum agents and have demonstrated antitumor activity in advanced solid tumors. Elimusertib (BAY1895344) is currently being tested in combination with cisplatin in urothelial cancer, and results have not been reported yet. Only a few studies have tested ATR inhibitors in the context of platinum resistance. Lung cancer cells deficient in *TP53* and *ERCC1* treated with an ATR inhibitor were resensitized to cisplatin by inducing replication catastrophe (17). In ovarian cancer cells, ATR inhibition increased the sensitivity to cisplatin through deregulation of its downstream kinases CHK1 and WEE1 and through inhibition of homologous recombination (18).

PKMYT1 is a crucial cell-cycle modulator as it phosphorylates and inhibits CDK1, preventing unscheduled mitotic entry (19–21). In *CCNE1*-amplified tumors, including TNBCs, a novel PKMYT1 inhibitor, RP-6306, was found to be synthetic lethal (22). PKMYT1 inhibition in combination with gemcitabine was shown to reverse resistance to endocrine therapy and palbociclib in ER^+^ breast cancers (23). Moreover, PKMYT1 inhibition can synergize with the alkylating agent temozolomide in glioblastoma (24). More recently, PKMYT1 inhibition was shown to synergize with cisplatin in osteosarcoma cells (25). A phase I clinical trial testing the PKMYT1 inhibitor RP-6306 with carboplatin and paclitaxel in *TP53*-mutated ovarian and uterine cancer (NCT06107868) is currently ongoing. Interestingly, the synthetic lethality of RP-6306 is also being evaluated in clinical trials with ATR inhibitors in solid tumors.

In the present study, we used patient-derived xenograft (PDX) cell lines (PDXC) from chemoresistant TNBCs and performed a pooled short hairpin RNA (shRNA)–based synthetic lethal screen that led to the identification of *ATR* as the top synthetic lethal target that resensitized TNBCs to carboplatin. We demonstrate that carboplatin synergizes with the ATR inhibitor BAY1895344 *in vitro* and that ATR inhibition can resensitize TNBC PDXs to carboplatin and also prevent tumor relapse. We also found that PKMYT1 inhibition can overcome carboplatin resistance in TNBCs. The ATRi/carboplatin and PKMYT1i/carboplatin combinations led to the accumulation of cells in G_2_–M and mitotic catastrophe. Together, these results highlight the vulnerability of TNBCs to cell-cycle regulators when combined with the DNA-damaging agent carboplatin, supporting further clinical investigation of these combinations in the context of patients with carboplatin-resistant TNBC.

## Materials and Methods

### Generation of PDXs and PDXCs

Written informed consent was provided by all patients included in this study for the generation of patient-derived models. PDXs originated from either primary breast tumors collected at surgery (T-830, T-817, T-786, PDX-1915, PDX-1735, and PDX-1939) or from skin metastases (BM-156 and BM-173). Prior treatments received by these patients with TNBC are shown in Supplementary Table S1. To generate PDXs, tumors were engrafted in the mammary fat pad of NOD-scid IL2Rγ^null^ (NSG) mice (The Jackson Laboratory). Once tumors reached 2,000 mm^3^, they were collected and serially passaged in new NSG mice or preserved as live tissue in FBS with 10% DMSO at −80°C for future engraftment.

PDXCs were generated from PDXs in-house at passages P0 to P1, in accordance with the Schlegel protocol (26). The biological sex of all cells generated is female. Cells were passaged every 2 to 5 days, depending on doubling time or when they reached 80% to 90% confluency, and fresh media was changed in between passages. Experiments were conducted using cells with passages under P25. Cells were cultured in a humid incubator with 5% CO_2_ at a temperature of 37°C. Authentication was confirmed with cytoscan to match the associated PDX and patient (Supplementary Fig. S1A) as well as mouse versus human PCR primers (Supplementary Fig. S1B). Additional information on PDXC generation, media, and validation can be found in the Supplementary Methods.

Cells were tested for *Mycoplasma* in the lab using the MycoAlert PLUS Mycoplasma Detection Kit (Lonza, cat. #LT07-518) according to the manufacturer’s instructions every month.

### Cell viability assay

Cells were seeded in 96-well plates and incubated at 37°C in a 5% CO_2_ humidified atmosphere. Twenty-four hours later, 100 μL of F medium containing the indicated supplements (Supplementary Table S2) was added. The media was removed 72 hours later and replaced with DMEM 10% alamarBlue solution (Invitrogen, cat. #DAL1100). Fluorescence was read on a FLUOstar OPTIMA (BMG Labtech), using 560-nm (excitation) and 590-nm (emission) filter settings.

For BM-156, given their poor adhesion, the sulforhodamine B (SRB) assay was used instead of alamarBlue because, with this method, cells were fixed with 10% trichloroacetic acid at 4°C before the removal of the medium at the end of the treatment (7 days). Cells were then rinsed with water before the addition of the SRB solution [0.2% SRB powder (Sigma, cat. #S1402) in 1% acetic acid] and incubated for 30 minutes at room temperature. The plates were rinsed with a 1% acetic acid solution, and Tris-base (pH 10.5) was added for 30 minutes at room temperature. Absorbance was measured on a FLUOstar OPTIMA.

Each condition was performed in triplicate. The percentage of cell viability was calculated by using the mean absorbance of the triplicate for a given condition normalized to the control or nontreated condition, which was set at 100% viability. Log dose-response curves were plotted with GraphPad (version 5.0), and the IC_50_ for each condition was calculated using the linear regression function, choosing a dose-response inhibition model (Supplementary Table S2).

### Combination index score

To determine if the combination of two drugs is synergistic, the Chou–Talalay combination index (CI) score was used (27). The formulation can be found in Supplementary Methods.

### Clonogenic assay

Cells were seeded in six-well plates and treated with the indicated treatments (Supplementary Table S2) 24 hours later. After 14 days, cells were fixed with 10% formalin (Fisher Scientific, cat. #SF100-4) and stained with 0.5% crystal violet (Sigma, cat. #HT90132-1L). Visible colonies were counted using a GelCount colony counter (Oxford Optronix).

### PDX clinical trials

Pieces (1 to 2 mm^3^) of live tissue were implanted into the mammary fat pad of 6- to 8-week-old NSG female mice for T-786, T-817, and BM-156 PDX models. Approximately 40 mice were engrafted, and tumor volume was measured every 2 days with an electronic caliper. Tumor volume was calculated using the following formula: (length × width^2^)/2. When tumors reached approximately 200 mm^3^, mice were randomized: vehicle (0.9% saline water), carboplatin 20 mg/kg intraperitoneally once a week, BAY1895344 40 mg/kg once a week (gavage), carboplatin + BAY1895344, AZD6738 25 mg/kg 5 days on, 2 days off (gavage), and carboplatin + AZD6738. Tumor volume was measured twice weekly. Mice were evaluated for signs of toxicity by monitoring body weight and general body condition. Mice were euthanized with CO_2_ and isoflurane when tumors reached the maximal tumor size permitted (2,000 mm^3^) or body weight loss was >20% of the initial body weight or there were other signs of animal distress or toxicity. The Studylog software (Studylog Systems) was used to facilitate data collection.

Smaller *in vivo* studies were also performed in additional PDX models (BM-173, PDX-1735, PDX-1886, PDX-1905, PDX-1915, PDX-1924, PDX-1939, PDX-1945, PDX-1971, PDX-1986, PDX-2076, and PDX-2089). Mice were randomized into three treatment groups (∼3/group): only vehicle, carboplatin 20 mg/kg once weekly, and carboplatin + BAY1895344 40 mg/kg once weekly. In cases where a complete tumor response was observed, treatment was interrupted to allow tumor regrowth. The tumor growth inhibition formula can be found in the Supplementary Methods.

### Annexin V/propidium iodide apoptosis assay

Cells were seeded in a six-well plate and treated 24 hours later as indicated (Supplementary Table S2). After 72 hours of treatment, floating dead cells were collected and combined with the trypsinized adherent cells. Apoptosis was measured using the BD Pharmingen FITC Annexin V Apoptosis Detection Kit (cat. #556547) according to the manufacturer’s instructions. Data were acquired on a FACS Canto II and analyzed with FACS Diva (BD Biosciences) or FlowJo software (Tree Star).

### Genetic screens

To identify novel genes in which inhibition confers sensitivity to carboplatin, we performed RNAi-based genetic screens. We used an shRNA “kinome” library targeting 535 human kinases and kinase-related genes (28, 29) and another shRNA library against ∼1,200 known target genes of clinically approved drugs. In brief, carboplatin-resistant cells were transduced with the shRNA libraries at low multiplicity of infection (MOI; ∼0.3) with a target of 1,000× coverage. Transduced cells were then selected in puromycin for 2 days, and a time 0 (T0) sample was collected. Transduced cells were then treated with vehicle or an IC_25_ of carboplatin (23.5 μmol/L) for 14 days, after which genomic DNA was extracted (time 1 or T1), and inserts were recovered with PCR amplification as described (30). The relative abundance of shRNAs in T1 treated with vehicle or with carboplatin was determined by next-generation sequencing analyzed by the model-based analysis of genome-wide CRISPR-Cas9 knockout (MAGeCK) statistical software package (version 0.5.8; ref. 31) to identify the top candidates resensitizing cells to carboplatin (31).

### siRNA and shRNA

Lentiviral transduction with low MOI was performed using the protocol described at https://portals.broadinstitute.org/gpp/public/resources/protocols. Briefly, 2.5 × 10^6^ HEK293T cells were seeded and transfected with the indicated lentiviral constructs, the packaging (psPAX2), and envelope (pMD2.G) plasmid by CaCl_2_. Virus-containing medium was collected for the transduction of stable cell lines. Following transduction, cells were selected with puromycin and/or blasticidin for 2 to 4 days and plated for downstream assays immediately after selection. Individual shRNA and open reading frame (ORF) vectors were from the MISSION TRC library (Sigma), and ORF collections were developed by members of the ORFeome Collaboration (Sigma–TransOMIC), provided by the McGill Platform for Cellular Perturbation of the Goodman Cancer Research Centre. Sequences can be found in Supplementary Table S3.

For siRNA transfection, cells were seeded at 200,000 cells per well in a six-well plate, and 48 hours later, they were transfected with siRNA using Lipofectamine RNAi/MAX (Thermo Scientific, cat. #13778075) following the manufacturer’s instructions. Information about the siRNAs can be found in Supplementary Table S3.

### Protein extraction and immunoblot analysis

Cells were lysed with RIPA buffer (Thermo Scientific, cat. #89900) supplemented with protease inhibitors aprotinin (10 μg/mL), leupeptin (10 μg/mL), phenylmethylsulfonyl fluoride (1 mmol/L), and the phosphatase inhibitors Na_3_VO_4_ (1 mmol/L) and NaF (5 mmol/L) and incubated for 15 minutes on ice. Cells were centrifuged at 13,000 rpm at 4°C, the supernatant was collected, and proteins were quantified with the Pierce BCA Protein Assay Kit (Thermo Scientific, cat. #23225). Membranes were blocked with 5% bovine serum albumin (Sigma-Aldrich, cat. #A1933) and incubated with primary antibodies (Supplementary Table S4) overnight at 4°C. Secondary antibodies were added the next day for 1 hour at room temperature. Enhanced chemiluminescence reagent was used to detect proteins (Sigma-Aldrich, cat. #GERPN2134 and cat. #GERPN2235).

### Immunofluorescence

Cells were seeded on circular cover slips (Fisher Scientific, cat. #12545102P) and treated 24 hours later with drugs (Supplementary Table S2). Cells were then washed with PBS, fixed, and permeabilized with PBS containing 0.1% Triton X-100 for 15 minutes, washed with PBS, and blocked with PBS containing 4% goat serum (Sigma-Aldrich, cat. #G9023). The coverslips were incubated for 1 hour and 30 minutes with a mouse primary antibody against γH2AX (Millipore, cat. #JBW301), diluted in PBS with 2% goat serum at 1:750, washed with PBS, and then incubated in PBS containing 2% goat serum for 1 hour with a goat secondary antibody raised against mouse, conjugated to Alexa Fluor 594 (Invitrogen, cat. #A11020), DAPI (Invitrogen, cat. #D3571), and Alexa Fluor 488 phalloidin (Invitrogen, cat. #A12379). The cells were washed before mounting the coverslips on slides. The slides were visualized using a ZEISS LSM800 confocal microscope with Airyscan. About 10 images per treatment group were unbiasedly acquired by selecting regions using the DAPI channel. Using Fiji software, a selection was made by contouring the cells with the phalloidin channel to then import this mask onto the Alexa Fluor 594 channel to measure the mean fluorescence intensity. Each treatment group contains at least 100 cells for each experiment, repeated 3 times.

### RT-qPCR

RNA extraction was done using the RNeasy Mini Kit (QIAGEN, cat. #74104) with RNase A (QIAGEN, cat. #19101) and DNase (QIAGEN, cat. #79254) according to the manufacturer’s instructions. The iScript cDNA synthesis kit (Bio-Rad, cat. #170-8891) was used to generate cDNAs according to the manufacturer’s instructions. Real-time PCR was performed using the SsoAdvanced Universal SYBR Green Supermix (Bio-Rad, cat. #172-5272). ATR mRNA quantification was normalized by measuring GAPDH mRNA. Primers can be found in Supplementary Table S3.

### Cell-cycle analysis

Cells were seeded in a 100 mm culture dish. After 48 hours, cells were treated with the indicated drugs and incubated at 37°C for 24 hours. Cells were then washed, trypsinized, centrifuged, resuspended in PBS, 5 mmol/L EDTA, and fixed for 30 minutes with 100% ethanol while vortexing at low speed. Cells were pelleted and washed again with PBS, 5 mmol/L EDTA and resuspended at 1 × 10^6^ cells/mL in PBS with 50 μg/mL propidium iodide and 20 μg/mL RNase A DNase-free. Cells were finally incubated at 37°C in the dark for 30 minutes and analyzed on a FACSCanto II instrument (Becton Dickinson). Results were analyzed using ModFit LT (Verity Software House, version 4.1.7).

### RNA sequencing of PDXCs

RNA was extracted using the RNeasy Mini kit (QIAGEN, cat. #74104) according to the manufacturer’s instructions. Triplicates for each condition were sequenced using the Illumina NextSeq 500 platform, 1 × 75 cycle High Output. RNA sequencing (RNA-seq) libraries were prepared from total RNA using poly(A) enrichment. Data analysis information can be found in Supplementary Methods.

### Statistics

Statistical analyses were performed using GraphPad software 5.0 (Prism). Data in figures are represented as mean ± SEM. For the bar graphs and the dot plot, statistical differences were assessed using the Student *t* test to compare two groups of interest. For tumor growth, statistical significance was assessed using the Student *t* test to compare two groups of interest at the end of the study. Mantel–Cox analysis of Kaplan–Meier curves was performed to analyze statistical differences in survival. Significance was considered with a *P* value <0.05: *, *P* < 0.05; ***, *P* < 0.01; and ***, *P* < 0.001.

### Study approval

The study was conducted in accordance with the Declaration of Helsinki and approved by the Institutional Review Board (or Ethics Committee) of the Jewish General Hospital (JGH). Patients consented to sample collection as part of the breast biobank of the JGH (protocol #05-006). PDX and PDXC generation was performed as part of protocol #14-168 approved by the JGH review ethics board. All animal studies were approved by McGill University’s Animal Care and Use Committee.

### Models availability

To promote transparency and ensure reproducibility, we are committed to sharing our PDX and PDXC models. This is subject to the approval of the institutional review ethics board.

## Results

### An shRNA screen in carboplatin-resistant TNBC PDX cells identifies ATR as a synthetically lethal gene with carboplatin

**Figure 1.**
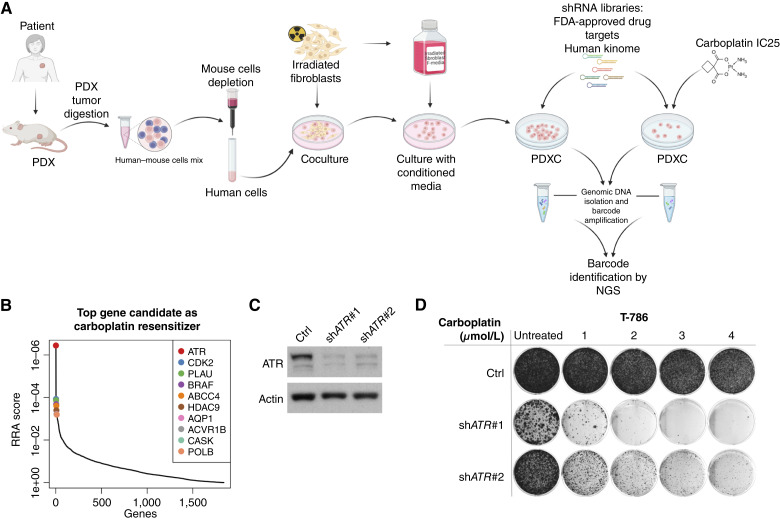
An shRNA screen reveals *ATR* as the top carboplatin sensitizer gene in a carboplatin-resistant TNBC PDXC (T786 PDXC). **A,** Schematic representation of the PDX and PDXC generation and the shRNA screen conducted to identify genes synthetically lethal with carboplatin. **B,** Plot of the top 10 carboplatin sensitizer genes rank-ordered by robust rank aggregation (RRA) scores calculated by MAGeCK, in which a smaller RRA score indicates more essentiality. The *ATR* gene appeared as the top carboplatin sensitizer gene in PDXC T-786. **C,** Immunoblot analysis of ATR in T-786 transduced with two independent *ATR* shRNAs. **D,** Clonogenic assay with the two *ATR* shRNA-transduced T-786 PDXCs. Cells were treated for 14 days with the indicated concentrations of carboplatin. NGS, next-generation sequencing. [**A,** Created in BioRender. Guay, J. (2026) https://BioRender.com/s50l814.]

To identify molecular vulnerabilities that can be therapeutically exploited to overcome resistance to carboplatin in TNBC, we performed a high-throughput shRNA screen using a carboplatin-resistant cell line model derived from a carboplatin-resistant PDX (T-786). The PDX model was generated from a patient who was resistant to carboplatin in both the neoadjuvant and metastatic settings. Tumor cells from the PDX were collected at an early passage to establish PDXCs using a conditionally reprogrammed cell protocol (26). PDXCs contain the same genomic changes as in both the original tumor and the PDXs from which they were derived (Supplementary Fig. S1A). *In vitro* resistance was confirmed by cell viability assays (IC_50_ = 52 μmol/L; Supplementary Fig. S2A; Supplementary Table S5). T-786 PDXCs were infected with pooled lentiviral shRNA libraries against FDA-approved drug targets (29) and the human kinome (∼1,700 druggable genes; ref. 28). Cells were cultured for 14 days with IC_25_ of carboplatin or vehicle, and the relative abundance of shRNA vectors identified by next-generation sequencing was quantified, followed by MAGeCK (Fig. 1A). The *ATR* gene was the top candidate in which silencing was synthetically lethal with carboplatin (Fig. 1B). Indeed, despite the known essential nature of *ATR* (32, 33), we observed that *ATR* silencing did not result in total loss of viability but in partial inhibition of proliferation (Supplementary Table S6; Supplementary Fig. S3A). In fact, when we treated T-786 PDXCs *ATR*-silenced cells with the ATR inhibitor BAY1895344 (elimusertib; Supplementary Fig. S3B and S3C), we found that the silenced cells were much more sensitive to ATR inhibition than the control siRNA cells, suggesting that residual ATR protein after silencing is required to maintain cell viability. Moreover, these ATR-silenced cells were significantly more sensitive to the fork-stalling agent aphidicolin (Supplementary Fig. S3E), supporting a greater sensitivity to replicative stress, in line with ATR’s role in controlling the replicative stress checkpoint. Therefore, although *ATR* silencing alone had an effect on viability and proliferation, its markedly greater effect on response to carboplatin established *ATR* as the top hit in our screen. The other four top targets identified included *CDK2*, *PLAU*, *BRAF*, and *ABCC4*. Of these, *CDK2* and *BRAF* are druggable but are clearly less potent in resensitizing to carboplatin than *ATR*. Indeed, *CDK2* silencing did not resensitize cells to carboplatin (Supplementary Fig. S4A and S4B). Moreover, *BRAF* is not mutated in this model.

### ATR inhibition resensitizes chemoresistant TNBC models to carboplatin

**Figure 2.**
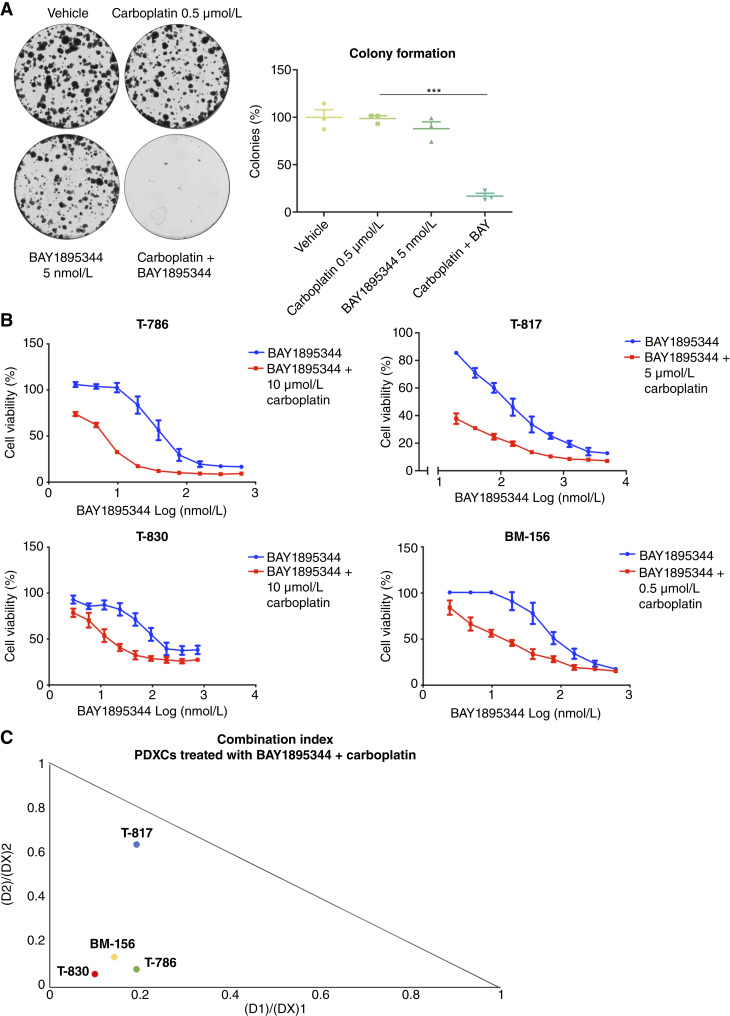
Pharmacologic inhibition of ATR synergizes with carboplatin in TNBC PDXCs. **A,** Representative images of the clonogenic assay of PDXC T-786 treated with vehicle, 5 nmol/L BAY1895344, 0.5 μmol/L carboplatin, and 5 nmol/L BAY1895344 + 0.5 μmol/L carboplatin (left) and the quantification (right) for 14 days, where a dot represents the relative density of cells compared with the vehicle for each biological replicate. Significance was assessed by the two-sided unpaired nonparametric Student *t* test. Mean ± SEM, *n* = 3, ***P < 0.001. **B,** Cell viability (%) of PDXCs T-786, T-817, T-830, and BM-156 measured by alamarBlue assay. Carboplatin in concentrations averaging between the IC_10_ and IC_25_ concentrations for each cell model was added to a gradient concentration of BAY1895344 (*n* = 3). **C,** Isobolograms representing the CIs of each TNBC PDXC treated with the combination of BAY1895344 and carboplatin.

To validate these results, we silenced *ATR* using two shRNAs in the same model (Fig. 1C). Although *ATR* silencing alone had little impact on the colony formation of T-786 cells, it strongly suppressed cell growth when combined with carboplatin (Fig. 1D). Similarly, *ATR* silencing with siRNAs resensitized T-786 PDXC to carboplatin in a cell viability assay (Supplementary Fig. S3D). Consistent with our RNAi results, the combination of the ATR inhibitor BAY1895344 (elimusertib) with carboplatin significantly reduced colony formation (Fig. 2A). Moreover, BAY1895344 synergized with carboplatin, using doses of carboplatin in the range of IC_10_ to IC_25_, in four PDXC models, all relatively carboplatin-resistant (Supplementary Fig. S2B–S2D), in cell viability assays (Fig. 2B and C). Additionally, carboplatin also synergized with subeffective doses of BAY1895344 (5 and 15 nmol/L), yielding CIs of 0.47 and 0.46, respectively (Supplementary Fig. S2E), consistent with a resensitization to carboplatin. We also tested a second ATR inhibitor, AZD6738 (ceralasertib), which was less potent than BAY1895344, with an IC_50_ >5-fold higher than BAY1895344 in T-786 (Supplementary Table S5). BAY1895344 yielded a stronger synergistic CI than AZD6738 in all PDXCs tested in combination with carboplatin (Fig. 2C; Supplementary Fig. S5A–S5C).

**Figure 3.**
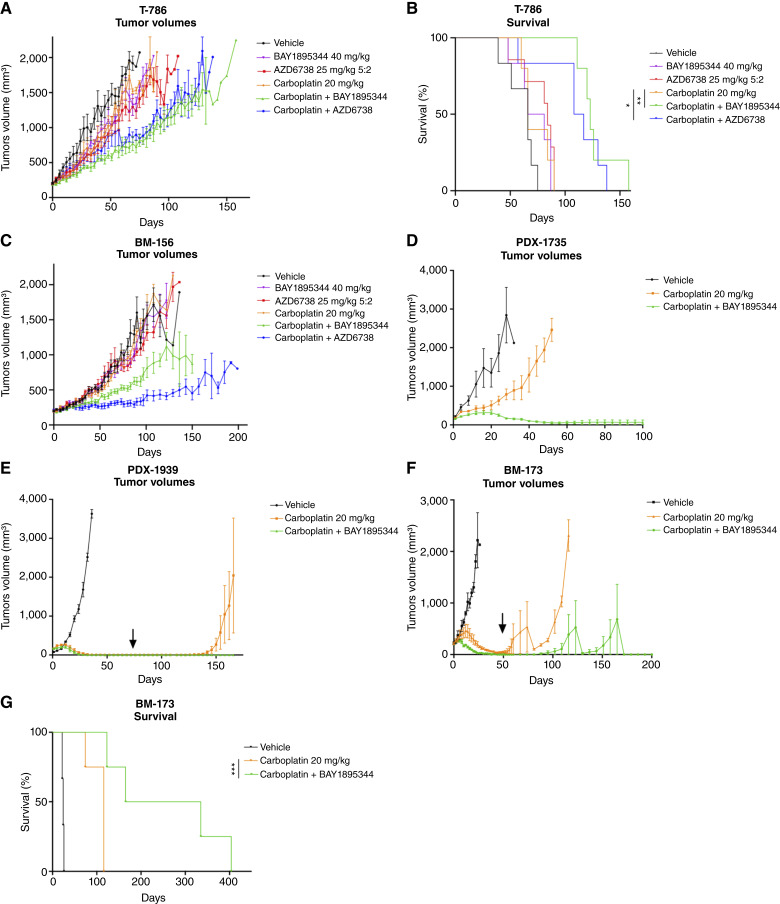
ATR inhibition resensitizes chemoresistant TNBC PDXs and TNBC PDXs that acquire resistance to carboplatin. **A,** Tumor growth and (**B**) survival in response to vehicle (*n* = 5), carboplatin 20 mg/kg once weekly (*n* = 7), BAY1895344 40 mg/kg once weekly (*n* = 6), AZD6738 25 mg/kg 5 days on, 2 days off (*n* = 6), carboplatin 20 mg/kg once weekly + BAY1895344 40 mg/kg once weekly (*n* = 6), or carboplatin 20 mg/kg once weekly + AZD6738 25 mg/kg 5 days on, 2 days off (*n* = 6) in PDX T-786. **C,** Tumor growth in response to vehicle (*n* = 5), carboplatin 20 mg/kg once weekly (*n* = 6), BAY1895344 40 mg/kg once weekly (*n* = 6), AZD6738 25 mg/kg 5 days, on 2 days off (*n* = 6), carboplatin 20 mg/kg once weekly + BAY1895344 40 mg/kg once weekly (*n* = 6), or carboplatin 20 mg/kg once weekly + AZD6738 25 mg/kg 5 days on, 2 days off (*n* = 6) in PDX BM-156. **D,** Tumor growth in response to vehicle (*n* = 3), carboplatin 20 mg/kg once weekly (*n* = 3), or carboplatin 20 mg/kg once weekly + BAY1895344 40 mg/kg once weekly (*n* = 3) in PDX-1735. **E,** Tumor growth in response to treatments (see **D**) in PDX-1939 (*n* = 3 per group). **F,** Tumor growth in response to treatments (see **D**) in PDX BM-173 (*n* = 4 per group). **G,** Survival in response to treatments (see **F**) in PDX BM-173 (*n* = 4 per group). Mean ± SD; ns, nonsignificant; *, *P* < 0.05; **, *P* < 0.01; ***, *P* < 0.001. Significance was assessed by unpaired nonparametric Mann–Whitney test of tumor size on the last day when the study included all individuals (tumor growth) or by the Mantel–Cox test for survival. Arrows represent where treatments stopped (**F** and **G**).

We next performed *in vivo* tests of the combination of carboplatin with both ATR inhibitors in two matching PDXs, from which the PDXCs were originally derived. We first determined that 20 mg/kg of carboplatin with 40 mg/kg of BAY1895344, both given once weekly, was well tolerated in NSG mice (Supplementary Fig. S6). In the T-786 model, both BAY1895344 and AZD6738 enhanced carboplatin efficacy, resulting in tumor growth inhibition (Fig. 3A). This was associated with improved survival for both combinations (Fig. 3B). In model BM-156, carboplatin combined with BAY1895344 or AZD6738 resulted in TGI of 40% and 65%, respectively (Fig. 3C). Collectively, these results support that ATR inhibition and carboplatin treatment offer a promising therapeutic avenue to treat carboplatin-resistant TNBC.

We screened an independent series of 12 TNBC PDX models for the efficacy of the ATRi/carboplatin combination compared with carboplatin alone using small (*n* = 3) experiments (Fig. 3D–G; Supplementary Fig. S7A–S7L). Of these, seven showed varying degrees of resistance to carboplatin, whereas five were sensitive (Supplementary Table S7). The addition of BAY1895344 to carboplatin resulted in tumor regression in PDX-1735 (Fig. 3D). Furthermore, we observed that acquired resistance to carboplatin was significantly delayed, if not prevented, by the addition of BAY1895344 in two carboplatin-sensitive PDX models, PDX-1939 and PDX BM-173 (Fig. 3E and F). Notably, PDX-1939 tumors never regrew even after treatment was stopped, whereas tumors regrew in the carboplatin arm. The average survival of BM-173 mice on the combination treatment was 4 times longer compared with carboplatin-treated mice (Fig. 3F). In a separate set of studies, BAY1895344 alone had no or only partial effect (PDX-1735) on tumor growth (Supplementary Fig. S7J–S7L). Together, these results confirm that combining ATR inhibition with carboplatin is a therapeutic strategy that can prevent tumor relapse in a subset of TNBC.

### Pharmacologic inhibition of ATR leads to a decrease in its protein expression

**Figure 4.**
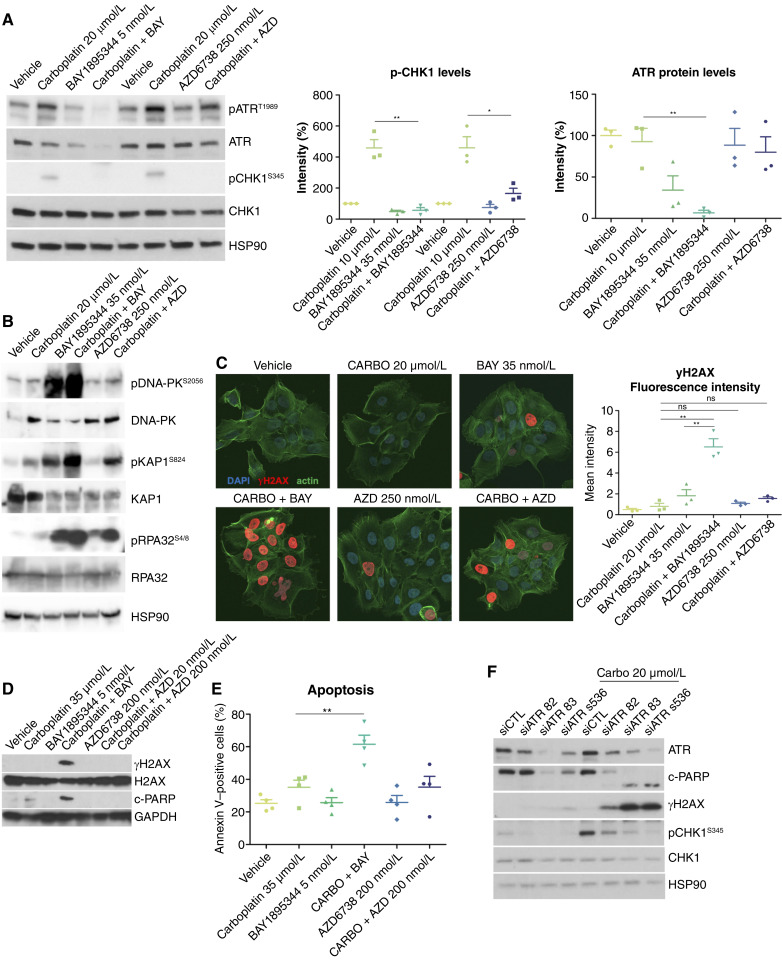
ATR inhibition and knockdown in carboplatin-treated TNBC PDXC induces DNA damage and apoptosis. **A,** Immunoblot analysis showing inhibition of ATR activation (p-ATR T1989) and downstream signaling (p-Chk1 S345) in response to 48 hours of the indicated treatments in PDXC T-786. Quantification of p-CHK1 (S345) and ATR total levels is shown on the right. Significance was assessed by the two-sided unpaired nonparametric Student *t* test. Mean ± SD, *n* = 3, ns, not significant; *, *P* < 0.05; **, *P* < 0.01. **B,** Immunoblot analysis showing stronger induction of the DNA-damage response (p-DNA-PK S2056 and p-KAP1 S824) and replicative stress markers (p-RPA32 S4/8) when ATR inhibitors are combined with carboplatin compared with carboplatin alone in PDXC T-786. **C,** Representative images (left) of IF of γH2AX levels (red) acquired by confocal microscopy and γH2AX levels quantification (right). Significance was assessed by the two-sided unpaired nonparametric Student *t* test. Mean ± SD; *n* = 3; ns, not significant; **, *P* < 0.01. Arrow shows the pan-nuclear γH2AX phenotype. **D,** Immunoblot analysis of DNA damage (γH2AX) and apoptotic marker (c-PARP) in response to the indicated treatments in PDXC T-786. **E,** Annexin V levels measured by flow cytometry in response to the indicated treatments in PDXC T-786. Significance was assessed by the two-sided unpaired nonparametric Student *t* test. Mean ± SD; *n* = 3; ns, not significant; **, *P* < 0.01. **F,** Immunoblot analysis showing ATR knockdown in PDXC T-786 treated with three independent siRNAs and the effect on downstream signaling (p-CHK1 S345), DNA damage (γH2AX), and apoptosis (c-PARP).

We next studied the effects of ATR inhibition on ATR signaling, DNA damage, and the cell cycle. ATR autophosphorylation was increased by carboplatin and completely abolished when BAY1895344 was added and decreased when AZD6738 was added (Fig. 4A). Once activated, ATR phosphorylates its primary effector CHK1 at Ser317 and Ser345 (34–36). In the T786 PDXC model, carboplatin alone resulted in CHK1 phosphorylation at both Ser317 and Ser345, and the addition of the ATR inhibitor resulted in a marked decrease in this CHK1 activation (Fig. 4A; Supplementary Fig. S8A). Interestingly, the combination of carboplatin with rabusertib, a CHK1 inhibitor, was not as synergistic as the combination with ATR inhibitors (Chou–Talalay CI, 0.88), suggesting that CHK1 may not be as critical as ATR for survival during carboplatin treatment of these resistant cells (Supplementary Fig. S9). Indeed, CHK1 did not emerge as a candidate in the original shRNA screen. These findings suggest that ATR signaling independent of CHK1 activation may be at least partly responsible for resistance to carboplatin in these cells.

Although carboplatin resulted in autophosphorylation of ATM, this activation was not abrogated when combined with ATR inhibitors, confirming the specificity of these ATR inhibitors (Supplementary Fig. S8B). We observed that ATR protein levels decreased with BAY1895344 alone and further when combined with carboplatin (Fig. 4A). This was also observed using another ATR antibody (Supplementary Fig. S8C) and in other TNBC PDXCs (Supplementary Fig. S8D). We did not find any involvement of transcriptional regulation or proteasomal degradation to explain this decrease in protein levels (Supplementary Fig. S8E–S8G). A cellular fractionation assay did not reveal ATR sequestration on the chromatin in response to the combination (Supplementary Fig. S8H). Although the mechanism underlying the suppression of ATR protein expression by BAY1895344 remains unclear, this unexpected effect may contribute to the effect of the BAY1895344 and carboplatin combination *in vitro*.

### Carboplatin in combination with ATR inhibitors leads to DNA damage, replicative stress, mitotic catastrophe, and cell death

We further studied the molecular effects of carboplatin–ATR inhibitor combination treatment in TNBC PDXCs. Carboplatin treatment alone resulted in a slight induction of p-RPA32, a marker of replicative stress (37–39) in PDXC T-786 (Fig. 4B). Treatment with BAY1895344 alone resulted in the induction of p-DNA-PK; p-KAP1, a substrate of ATM induced by double-strand breaks (40); and p-RPA32, which was exacerbated by the addition of carboplatin. To measure levels of γH2AX, the classic DNA damage marker (41), we used two methods: immunofluorescence (IF) and Western blot. The pattern of IF staining can be that of discrete foci or uniform pan-nuclear staining, the latter reflecting excessive replicative stress, mitotic catastrophe, and cell death (39). We detected high baseline levels of discrete γH2AX foci in PDCX T-786, which were not significantly increased with carboplatin treatment (Fig. 4C; Supplementary Fig. S10A and S10B). Upon treatment with BAY1895344, we observed a shift in the γH2AX staining pattern from discrete foci to a uniform pan-nuclear distribution, and more so with the combination of BAY1895344 and carboplatin. Western blot analysis also confirmed the induction of γH2AX with BAY1895344, which then increased with the combination treatment (Supplementary Fig. S10C). These findings support that ATR inhibition induces a γH2AX response that is further enhanced when combined with carboplatin. After 3 days of treatment, these cells started to undergo apoptosis as evidenced by increased levels of cleaved PARP and annexin V (Fig. 4D and E). Higher doses of AZD6738 could initiate a similar response (Supplementary Fig. S10C), pointing to a lower potency. To assess whether extensive γH2AX induction was a consequence of apoptosis or occurred before apoptosis, we treated cells with a combination of carboplatin and BAY1895344 in the presence of increasing concentrations of the irreversible pan-caspase inhibitor Z-VAD(OH)-FMK. γH2AX levels remained elevated despite caspase inhibition, indicating that its induction is not a downstream consequence of apoptosis (Supplementary Fig. S10D). Genetic inhibition of *ATR* using three different siRNAs also showed DNA damage and apoptosis, as indicated by γH2AX and cleaved PARP in carboplatin-treated cells (Fig. 4F). Altogether, these results show that both pharmacologic and genetic ATR inhibition enhance carboplatin-induced DNA damage and replicative stress, leading to apoptosis. This supports prior findings in various models, including TNBC (11, 42), and highlights the essential role of ATR signaling in the survival of carboplatin-treated T-786 cells.

**Figure 5.**
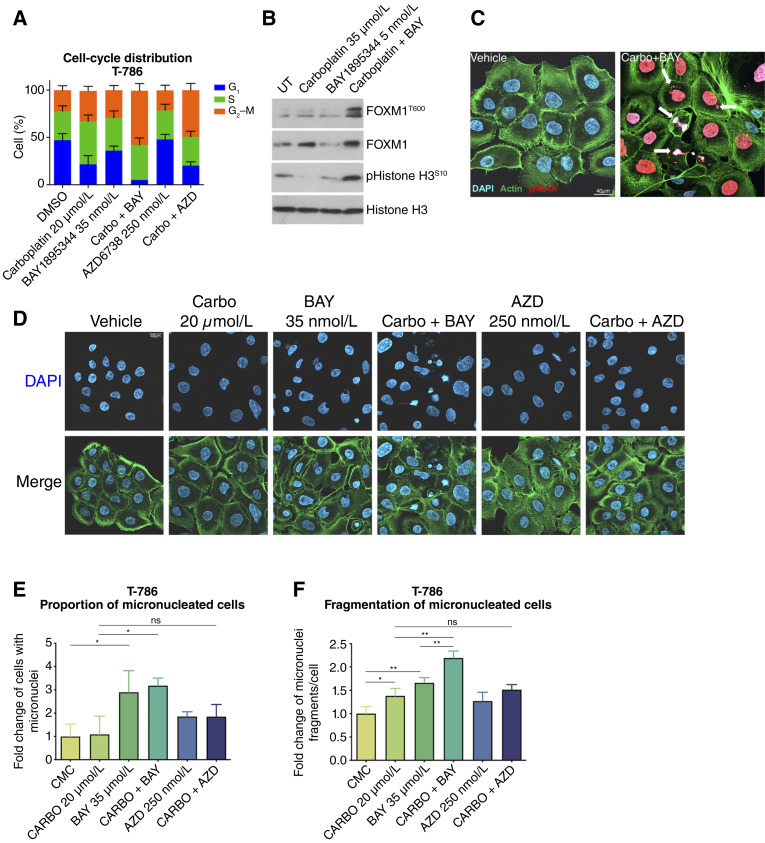
Combination of carboplatin and ATR inhibitors leads cells to mitotic catastrophe. **A,** Cell-cycle distribution performed by flow cytometry in response to 48 hours of indicated treatments in T-786 PDXC. **B,** Immunoblot showing the phosphorylation of the mitotic program transcription factor FOXM1 and histone H3 in response to 48 hours of indicated treatments. **C,** Representative images showing T-786 PDXC stained for γH2AX (red) in response to vehicle compared with the combination of carboplatin (20 μmol/L) with BAY1895344 (35 nmol/L). Arrows indicate micronuclei, cells undergoing mitosis (regardless of DNA damage), and failed mitosis. **D,** Representative images showing T-786 PDXC stained with DAPI (blue, top and bottom) and phalloidin (green, bottom), comparing the different treatment arms and showing micronucleation and nuclear fragmentation in response to BAY1895344 and the combination. **E,** Quantification of the proportion of cells with micronuclei in response to 48 hours of indicated treatments. Significance was assessed by the two-sided unpaired nonparametric Student *t* test. Mean ± SD; *n* = 3; ns, not significant; *, *P* < 0.05. **F,** Quantification of the number of fragments per cell with micronuclei in response to 48 hours of indicated treatments. Significance was assessed by the two-sided unpaired nonparametric Student *t* test. Mean ± SD; *n* = 3; ns, not significant; *, *P* < 0.05; **, *P* < 0.005.

It is well established that ATR activation can trigger cell-cycle arrest in S-phase by CHK1-dependent inhibition of CDC25A phosphatase activity on CDK2, whereas ATR inhibition abrogates this checkpoint and allows cell-cycle progression despite DNA damage (9, 10, 43, 44). Recent evidence shows that ATR controls the S–G_2_ checkpoint by delaying FOXM1 phosphorylation until G_2_, thus inhibiting the transcription of mitotic gene networks (9). In PDXCs T-786, ATR inhibition alone did not result in any appreciable change in cell-cycle dynamics. On the other hand, carboplatin alone resulted in an increase in the number of cells in S-phase and G_2_–M-phase (Fig. 5A), consistent with an increased time for repair of carboplatin-induced DNA damage before mitosis. When ATR was inhibited, the addition of carboplatin resulted in a marked accumulation of cells in G_2_–M, suggesting that ATR prevents S–G_2_ progression in carboplatin-treated, carboplatin-resistant cells (Fig. 5A). Histone H3 was phosphorylated in response to the combination treatment, indicating mitotic entry, as was FOXM1, the master regulator of the mitotic gene network expression, indicating its activation (Fig. 5B). We also observed mitotic figures in cells treated with the combination despite excessive γH2AX, together with nuclear fragmentation (Fig. 5C) and micronucleation (Fig. 5D), signs of premature mitosis resulting in mitotic catastrophe (45). Indeed, more cells treated with carboplatin and BAY1895344 contained micronuclei (Fig. 5E), whereas the number of micronuclei per cell was slightly increased by carboplatin or BAY1895344 alone and more so by the combination (Fig. 5F). These mitotic defects are suggestive of a mitotic catastrophe triggered by the combination of carboplatin with ATR inhibition (46, 47).

### ATR inhibition combined with carboplatin induces the transcription of mitotic processes

To better understand the effect of combining BAY1895344 with carboplatin, we performed RNA-seq of T-786 cells after 24 hours of treatment. Treatment with carboplatin alone resulted in the significant differential expression of only 45 genes, of which 39 had their expression suppressed (adjusted *P* < 0.05; Supplementary Fig. S11). Remarkably, 37 of these genes are regulated by the FOXM1 transcription factor, according to the ChEA transcription factor targets dataset (48). A gene ontology analysis (GOA) of the biological processes of these differentially expressed genes revealed an enrichment for upregulated genes involved in the negative regulation of mitosis and for downregulated genes involved in chromosome segregation and mitosis (Supplementary Fig. S12A and S12B). These genes included *CCNB1*, *CCNB2*, *CDC20*, several *KIF*s, *AURKA*, and *PLK1*. We then compared the transcriptome of these cells treated with carboplatin alone with that of the combination treatment with carboplatin and 5 nmol/L BAY1895344 for 24 hours. DESeq2 analysis revealed that 746 genes were significantly (adjusted *P* < 0.05) upregulated or downregulated. Gene ontology analysis revealed that the combination treatment resulted in a significant enrichment for biological processes related to chromosome segregation and mitosis for upregulated genes (Supplementary Fig. S12C), whereas downregulated genes were enriched for the biological processes of DNA replication and cell-cycle control (Supplementary Fig. S12D). Remarkably, all 39 genes that had been suppressed by carboplatin treatment alone were now significantly upregulated by the addition of an ATR inhibitor, consistent with our observation of FOXM1 phosphorylation with the combination. These findings support that T-786 cells resist carboplatin by inhibiting mitosis and cell division and that this program is tightly regulated by ATR. Moreover, this further supports our findings that mitotic entry is triggered when combining the ATR inhibitor with carboplatin. On the other hand, processes related to DNA replication and cell-cycle control were downregulated in response to the combination, suggesting ATR plays an essential role in suppressing replication and slowing the cell cycle to allow for the repair of carboplatin-induced DNA damage in these carboplatin-resistant cells.

### Inhibition of PKMYT1 resensitizes cells to carboplatin

**Figure 6.**
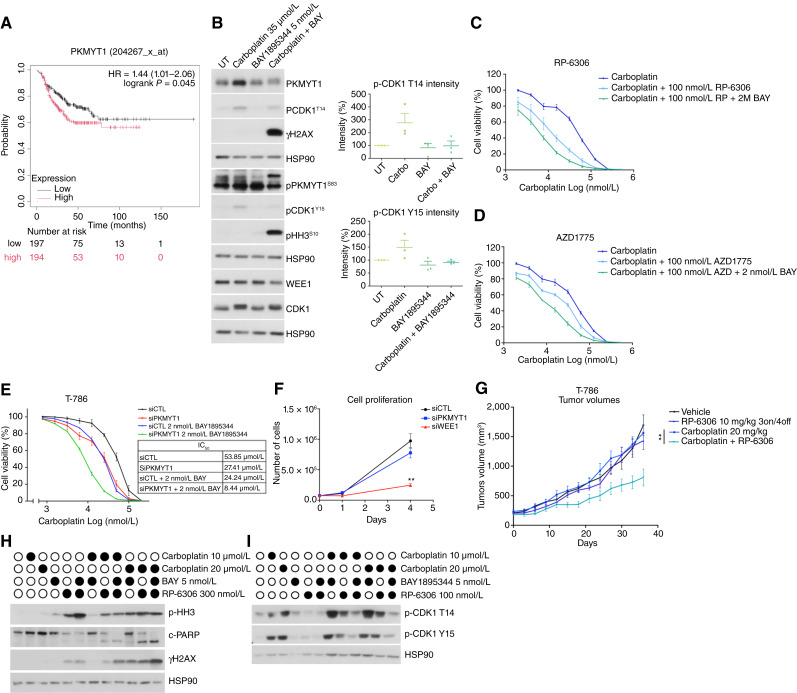
PKMYT1 inhibition enhances carboplatin sensitivity through CDK1 activation and increased DNA damage in TNBC PDX models. **A,** Kaplan–Meier analysis for overall survival of patients with chemotherapy-treated TNBC in the Kaplan–Meier plotter cohort with high or low levels of PKMYT1. **B,** Immunoblot analysis showing CDK1 regulation, DNA damage (γH2AX), and mitotic entry (pHH3). CDK1 phosphorylation (T14 and Y15) quantification is shown on the right (*n* = 3). **C** and **D,** Cell viability (%) of PDXC T-786 measured by alamarBlue assay after treatment with the indicated drug combinations (*n* = 3). **E,** Cell viability (%) of PDXC T-786 transfected with a pool of *PKMYT1* siRNAs measured by alamarBlue in response to the indicated treatments. IC_50_ values are presented on the right (*n* = 3). **F,** Cell proliferation of PDXC T-786 transfected with *PKMYT1* or *WEE1* siRNAs (*n* = 3). The number of cells after 24 hours and 4 days after plating was determined by manual cell counting. **G,** Tumor growth in response to the indicated treatments in PDX T-786. Randomized mice were treated with vehicle; carboplatin 20 mg/kg once weekly; RP-6306 10 mg/kg twice a day, 3 days on, 4 days off; or the combination of carboplatin and RP-6306. Each treatment arm contains 4 to 6 mice. **, *P* < 0.01. Significance was assessed by unpaired nonparametric Mann–Whitney test of tumor size. **H,** Immunoblot analysis showing c-PARP, p-HH3, and γH2AX levels in response to 48 hours of treatments with the indicated drug combinations in PDXC T-786. **I,** Immunoblot analysis showing CDK1 phosphorylation in response to the indicated treatments in PDXC T-786.

Mitotic entry is triggered by CDK1 (20) complexing with cyclin B1 (49, 50), a process regulated by the inhibitory activity of PKMYT1 and WEE1 through their phosphorylation of Thr14 and Y15 on CDK1. In fact, PKMYT1 can phosphorylate both Thr14 and Y15 (51, 52), whereas WEE1 only phosphorylates Y15 (53). PKMYT1 is itself phosphorylated in mitosis, which results in its inactivation (52, 54, 55). Interestingly, high expression of PKMYT1 is associated with poor prognosis in patients with chemotherapy-treated TNBC (Fig. 6A).

Treatment with carboplatin resulted in increased expression of PKMYT1, but not WEE1, and increased inhibitory phosphorylation of CDK1, suggesting a critical role for PKMYT1 in the regulation of the delay in mitotic entry induced by carboplatin in T-786 (Fig. 6B). Although ATR inhibition alone did not alter the expression or activation of PKMYT1 or CDK1, the combination of BAY1895344 with carboplatin resulted in a marked decrease in PKMYT1 protein levels compared with carboplatin alone. Additionally, the combination led to the phosphorylation of PKMYT1 (Fig. 6B). PKMYT1 has been shown to be hyperphosphorylated in mitosis (21, 54). The kinases responsible for this phosphorylation are not well documented in humans, but studies in other organisms suggest it could be AKT1 (56), MEK1 (57), p90RSK (58), and PLK1 (55). Interestingly, *PLK1* is one of the genes transcriptionally downregulated by carboplatin treatment, and its expression is upregulated with the addition of BAY1895344 (Supplementary Fig. S11). We also observed a decrease in the inhibitory T14 and Y15 CDK1 phosphorylation with the combination, consistent with the progression of the DNA-damaged cells into mitosis, as confirmed by the concomitant increase in γH2AX and pHH3 (Fig. 6B). This is in line with recent findings that identified CHK1 as a regulator of PKMYT1 through its phosphorylation at S143 (59). Altogether, these results are consistent with PKMYT1’s role in delaying mitosis in response to carboplatin, a function that seems to be decreased when the ATR inhibitor is added.

To better understand the implications of PKMYT1 in carboplatin resistance, we tested the novel PKMYT1 inhibitor, RP-6306 (60, 61), in combination with carboplatin using a cell viability assay. A clear synergistic effect was observed in PDXC T-786 (CI = 0.52), and the addition of low doses of BAY1895344 further resensitized the cells to carboplatin (Fig. 6C). These results were further validated in the other three PDXCs in which varying degrees of synergy were observed for the RP-6306 and carboplatin combination: PDXC T-817 (CI = 0.53), T-830 (CI = 0.85), and BM-156 (CI = 0.60; Supplementary Fig. S13A–S13C). In contrast, the WEE1 inhibitor AZD1775 (adavosertib) showed only mild synergy with carboplatin in PDXC T-786, with a CI close to 0.9 (CI = 0.86; Fig. 6D). Adavosertib did inhibit WEE1, as shown by the decrease in CDK1 phosphorylation on Y15 when combined with carboplatin (Supplementary Fig. S14A). Using siRNA silencing of *PKMYT1*, we found a 2- to 3-fold increase in sensitivity to carboplatin in *PKMYT1*-silenced PDXC T-786 and a 6- to 7-fold increase when a low concentration of BAY1895344 was further added (Fig. 6E; Supplementary Fig. S14B). Similar effects were observed with *WEE1* RNAi silencing (Supplementary Fig. S14B and S14C). However, we noticed a marked effect of the *WEE1* knockdown alone on proliferation, contrary to *PKMYT1* silencing alone, which showed no effect on proliferation (Fig. 6F). Given the promising effect of RP-6306 with carboplatin *in vitro*, we validated this combination *in vivo* in the matching PDX T-786. RP-6306 had no effect alone, but the combination with carboplatin significantly delayed tumor growth (Fig. 6G).

As both ATR and PKMYT1 seem to be involved in carboplatin resistance in our models, we investigated whether a triple combination of BAY1895344, RP-6306, and carboplatin could be more efficient at overcoming carboplatin resistance than the double combinations. Indeed, the triple combination led to higher levels of DNA damage and apoptosis than the dual combination of carboplatin with RP-6306 or with BAY1895344 (Fig. 6H). This correlated with a greater decrease in CDK1 phosphorylation on both T14 and Y15 with the triple combination compared with both double combinations (Fig. 6I). These findings were further supported by *PKMYT1* and *ATR* double knockdown via siRNAs, which led to increased mitotic catastrophe when combined with carboplatin (Supplementary Fig. S15). Altogether, these results support that PKMYT1 contributes to carboplatin resistance by controlling mitotic entry and that PKMYT1 may be an effective target for carboplatin-resistant TNBC cells.

We studied the on-treatment transcriptional changes upon the addition of RP-6306 to carboplatin to compare them with those described above for the BAY1895344/carboplatin combination. The differentially expressed genes were somewhat different. Treatment with RP-6306 reversed the carboplatin-induced suppression of 26 of the aforementioned 39 genes suppressed by carboplatin, compared with the reversal of all of them with ATR inhibition (Supplementary Table S8). GOA of the upregulated genes revealed an enrichment for genes associated with ribosome biogenesis and translation (Supplementary Fig. S12E), whereas the downregulated genes were enriched for cell adhesion, cell-cycle control, and DNA replication (Supplementary Fig. S12F).

### Transcriptional profiling of responders and nonresponders PDXs

To better understand which subset of TNBC might respond to the ATRi/carboplatin combination and to identify candidate biomarkers predictive of response to the combination, we investigated the genomic and transcriptional differences between models that favorably responded to the combination treatment compared with carboplatin alone. The four models identified as responders were PDX T786, PDX BM-156, PDX-1735, and PDX-1939. PDX-1939 initially showed regression with both carboplatin and the combination but later regrew only with carboplatin and not with the combination, suggesting that the combination prevented the acquisition of carboplatin resistance. It was therefore classified as a responder. In contrast, the six nonresponders were PDX-1915, PDX-1924, PDX-1971, PDX-1905, PDX-2076, and PDX-2089 (Supplementary Table S7). Whole-genome sequencing, SNP–comparative genomic hybridization (CGH), and whole-exome sequencing were performed on these models. We also focused our attention on 70 genes selected for their role in the cell cycle, DNA damage repair, and ATR response (62–65). Interestingly, 1 of these 10 PDX models contained a missense variant of *ATM*: PDX-1971 (p.Asp2720Asn; Supplementary Fig. S16; ref. 66), but this model was not responsive to the ATRi/carboplatin combination. A *BRCA1* mutation was detected in one of the responders and a *BRCA2* mutation in one of the nonresponders. As expected, there were few other mutated genes besides *TP53*. Copy number changes were inferred from whole-genome sequencing and SNP-CGH (CytoScan) and did not reveal obvious differences between responders and nonresponders, except for two of the four responder tumors showing an increase in the copy number of the *FOXM1* gene. These copy-number changes were not focal amplifications however.

**Figure 7.**
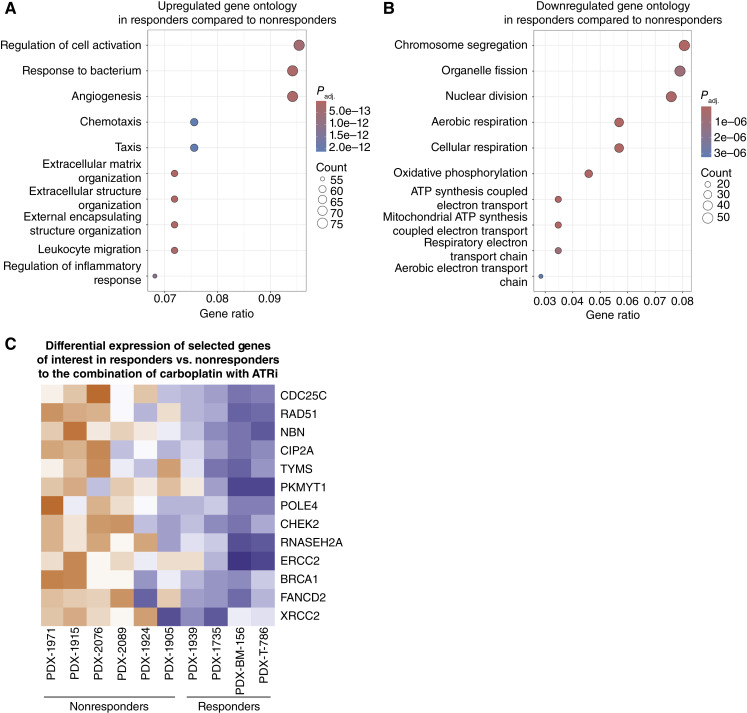
Transcriptional downregulation of cell-cycle genes identifies a vulnerability to carboplatin and BAY1895344 combination. **A,** Gene ontology analysis of upregulated and (**B**) downregulated genes in responders compared with nonresponders. **C,** Heatmap representing the significant transcriptional modulation genes from the gene list used for the model characterization. The represented genes were selected with *P* < 0.05.

We performed RNA-seq analysis on all 10 PDX models. Principal component analysis revealed distinct clustering of responders and nonresponders, highlighting clear transcriptional differences between these groups (Supplementary Fig. S17). DSeq2 analysis was performed between responders and nonresponders. Gene ontology analysis indicated that responders exhibited higher expression of genes associated with cell activation, response to bacterium, and angiogenesis (Fig. 7A). The responders also exhibited lower expression of genes associated with chromosome segregation, organelle fission, and nuclear division (Fig. 7B). We also examined the expression of the selected group of 70 genes (Supplementary Fig. S16). Notably, 13 of these 70 genes had significantly lower RNA levels in responders relative to nonresponders (Fig. 7C). These included the cell-cycle regulator *CDC25C*, key DNA damage repair genes *RAD51*, *BRCA1*, *XRCC1*, *NBN*, *CHEK2*, *FANCD2*, and *ERCC2*, as well as *PKMYT1*. Altogether, these findings suggest that gene expression variation in DNA repair and cell-cycle processes may influence the response to the combination of carboplatin and ATR inhibitors. Furthermore, low expression of PKMYT1 may be a potential biomarker for predicting response to this combination therapy, concordant with the increased effects of silencing both *PKMYT1* and *ATR* in reversing carboplatin resistance.

## Discussion

Carboplatin is one of the mainstays of chemotherapy treatment for patients with TNBC. This DNA-damaging agent acts primarily by binding to DNA and producing inter- and intrastrand cross-links (67). Although carboplatin is highly effective in both early and late TNBC, resistance inevitably develops in more advanced contexts. Many mechanisms of resistance have been elucidated, including alterations in DNA repair mechanisms, which ensure the continued viability of the cell (68).

We have generated clinically relevant patient-derived models of carboplatin resistance, including PDX and PDXCs, from drug-resistant TNBCs and found through a shRNA screen that *ATR* was the top gene in which inhibition led to carboplatin resensitization. We showed that two ATR inhibitors, namely AZD6738 and BAY1895344, can resensitize our PDXCs and PDXs to carboplatin *in vitro* and *in vivo*, highlighting the potential for ATR inhibition to overcome carboplatin resistance. Our observation that tumors from PDX-1939 did not regrow following the cessation of treatment is particularly striking, suggesting that the combination therapy may induce long-lasting responses that prevent relapse. Our exploratory analysis of PDXs responding or not responding to the ATR inhibitor/carboplatin combination suggests that the expression levels of specific genes involved in cell-cycle regulation and DNA damage repair may serve as valuable biomarkers for predicting the efficacy of this combination.

Interestingly, although both ATR inhibitors target the same kinase domain, they showed some differences in tumor response in our models (e.g., differential response in BM-156 tumors, Fig. 3C), as well as in their effects on DNA damage repair factors (e.g., Fig. 4B). Differences between the two ATR inhibitors used in this study are further underscored by the observation that ATR protein loss occurred only in response to BAY1895344 *in vitro*. We excluded proteasomal degradation and transcriptional regulation as causes of this loss, suggesting that alternative posttranscriptional mechanisms, such as impaired translation, may be involved. For instance, in *Arabidopsis thaliana*, a stable G-quadruplex structure in the 5′-untranslated region of *ATR* mRNA has been shown to inhibit its translation without affecting transcription (69). This effect, observed only with BAY1895344 and not AZD6738, may contribute to its greater potency. Additionally, BAY1895344 led to stronger induction of p-DNA-PK, which could reflect more severe replication stress, greater ATR suppression, or off-target modulation of DNA-PK signaling. Together, these findings suggest that although both compounds inhibit ATR, differences in potency, ATR protein stability, and downstream signaling account for their distinct cellular effects. Further investigation is warranted to determine whether these mechanisms also occur *in vivo*.

ATR acts to delay cell-cycle progression via an intra-S or G_2_–M checkpoint allowing greater time for the repair of DNA damage and for the resolution of replicative stress (45, 70, 71). More recently, ATR has also been implicated in an S–G_2_ checkpoint, in which it can prevent the transcription of mitotic genes via CDK1 and FOXM1, further preventing early mitotic entry (9). Our results indicate that ATR inhibition with carboplatin increases DNA damage in carboplatin-resistant cells, leading to excessive replicative stress and subsequent apoptosis. Our data also revealed that ATR inhibition alone did not significantly alter the cell cycle, whereas the combination with carboplatin led to the accumulation of cells in the G_2_–M-phase. This supports ATR’s role in critical checkpoint control that normally prevents cells with damaged DNA from entering mitosis. In fact, we observed an increased presence of mitotic figures, micronucleation, and nuclear fragmentation, further supporting the notion that the combination treatment results in premature mitotic entry, leading to mitotic catastrophe. PDXC T-786 treated with a combination of ATR inhibition and carboplatin showed high levels of γH2AX concurrently with high levels of p-HH3, suggesting mitotic entry, whereas DNA damage remains unrepaired.

These events are suggestive of mitotic catastrophe occurring in the combination-treated cells. We acknowledge that the definition of mitotic catastrophe remains somewhat ambiguous. The term refers to a mechanism of cell fate characterized by abnormally segregated chromosomes observed during mitosis, which renders these cells “mitosis-incompetent.” Mitotic catastrophe is due to the premature entry into mitosis of cells with unrepaired DNA or aberrant chromosomal anomalies, leading to an accumulation of cells in G_2_–M (“mitotic arrest”) and likely cell death. In our combination-treated cells, we observed G_2_–M arrest and cell death coincident with the appearance of morphologically abnormal nuclei and mitotic figures. Indeed, we are not the first to show mitotic catastrophe with ATR inhibition, as it was observed in ATR- and TP53 (70)-deficient leukemic cells (14). We did not perform short-term live cell imaging to help support this although our morphologic observation, together with the presence of DNA damage and mitotic arrest, is compelling evidence (45, 70).

We found that the combination of carboplatin with ATR inhibition results in FOXM1 phosphorylation, facilitating mitotic entry. RNA-seq data from our PDXC models treated with carboplatin or carboplatin and ATR inhibition support the role of FOXM1, in which target genes involved in chromosome segregation and mitosis are suppressed by carboplatin treatment, delaying mitotic entry. Adding ATR inhibition prevents this suppression, activating mitotic entry. Mitotic entry was further demonstrated by a decrease in the inhibitory phosphorylations of CDK1 (T14 and Y15) in the combination treatment compared with its induction by carboplatin alone. ATR can regulate CDK1 via its effector CHK1 that can inhibit the CDC25 phosphatases, which are responsible for removing these inhibitory phosphorylations (43, 44, 71). We found that the knockdown of either kinase responsible for phosphorylating CDK1, *WEE1*, and *PKMYT1* could also resensitize PDXC T-786 to carboplatin, highlighting the pivotal role of the G_2_–M checkpoint in carboplatin resistance in TNBCs. Contrary to *PKMYT1* knockdown, *WEE1* knockdown had a drastic effect on cell proliferation. This is in line with PKMYT1 being important in halting the cell cycle in response to DNA damage while being dispensable for unperturbed cell cycling (21). The differential effects observed suggest that targeting PKMYT1 with carboplatin could provide a more specific approach to sensitize resistant tumors without substantially impairing normal cell proliferation.

It is noteworthy that neither *PKMYT1* nor *WEE1* emerged as hits in our shRNA screen, which was conducted using a clonogenic assay design. This low-density setup may favor the expansion of specific subpopulations, particularly within our heterogeneous PDXC model. In contrast, *ATR* was a robust hit, sensitizing carboplatin-resistant cells both in the screen and under short-term, high-density conditions. Nevertheless, PKMYT1 inhibition effectively resensitized resistant TNBC to carboplatin *in vivo*, supporting its relevance as a therapeutic target. On the other hand, *CDK2* was also identified as a hit in the screen (Fig. 1B), but it did not resensitize to carboplatin in short-term assays (Supplementary Fig. S17).

Interestingly, carboplatin treatment increased PKMYT1 expression, and high levels of PKMYT1 are associated with poor prognosis in patients with TNBC treated with chemotherapy, suggesting it may play a role in resistance. Moreover, we observed phosphorylation of PKMYT1 in response to the combination of carboplatin with BAY1895344, implying that ATR can regulate this inhibitory phosphorylation. This is in line with a recent publication that highlighted the role of CHK1 in phosphorylating and inhibiting PKMYT1 (59).

PKMYT1 has recently gained attention as a promising target although its therapeutic exploration was previously limited by the lack of specific inhibitors. Using the novel PKMYT1 inhibitor RP-6306 (60), we were able to resensitize carboplatin-resistant models, highlighting the potential of PKMYT1 inhibition as a therapeutic strategy to enhance the efficacy of carboplatin in drug-resistant TNBCs. Although treatment with RP-6306 was able to partially reverse the carboplatin-induced gene suppression program, other genes were transcriptionally altered in the RP-6306/carboplatin combination, warranting further investigation to understand their role in mediating the synergistic effects with carboplatin. Currently, RP-6306 is being tested in clinical trials in combination with carboplatin and paclitaxel for ovarian and uterine cancers (NCT06107868) and with the ATR inhibitor RP-3500 for advanced solid tumors (NCT04855656). Although our results demonstrate the potential of a triple combination in effectively combating carboplatin resistance in TNBC, this triple combination may be overly toxic.

Preclinical investigation of RP-6306 has been, in part, conducted in the context of *CCNE1* amplification or *FBXW7* loss (61) and, more recently, in CDK4/6-resistant ER^+^ breast cancer cells (23). Notably, none of our PDXCs harbor these alterations (Supplementary Fig. S16), underscoring the need to further investigate the mechanisms underlying RP-6306 efficacy in TNBC. A key limitation of this study is the absence of a defined biomarker predicting response to the combination of carboplatin with either ATR or PKMYT1 inhibitors. Nonetheless, our findings highlight cell-cycle disruption as a promising therapeutic strategy in carboplatin-resistant TNBC and suggest that downregulation of mitotic processes may underlie this vulnerability.

In conclusion, a subset of carboplatin-resistant TNBCs relies on ATR and PKMYT1 to slow the cell cycle and buy time for the repair machinery to handle DNA damage and replicative stress. Our findings suggest clinical value for the combination of ATR or PKMYT1 inhibition with carboplatin in carboplatin-resistant TNBCs. Future studies should explore the clinical applicability of these combination therapies in patients with resistant tumors, guided by biomarkers that can predict response and improve personalized treatment strategies in TNBCs.

## Supplementary Material

Supplementary Table S1 PDX origins

Supplementary Table S2 Drug concentrations

Supplementary Table S3 shRNA, siRNA and primers sequences

Supplementary Table S4 List of antibodies

Supplementary Table S5 PDX responses to carboplatin and the carboATRi combination

Supplementary Table S6 Results of pooled shRNA ATR screen

Supplementary Table S7 PDX responses to carboplatin and combination therapy

Supplementary Table S8 Differentially expressed genes in response to carboplatin

Supplementary Figure S1 Genomic Fidelity of PDX models

Supplementary Figure S2 Carboplatin resistant PDXs

Supplementary Figure S3 ATR knockdown impairs proliferation of PDXC T-786 cells.

Supplementary Figure S4 CDK2 silencing results

Supplementary Figure S5 Combination AZD6738 and carboplatin

Supplementary Figure S6 Toxicity of ATR inh and carboplatin

Supplementary Figure S7 TNBC PDX small trials

Supplementary Figure S8 Effect of ATR inhibition on ATM-Chk2 pathway and ATR protein/mRNA levels in TNBC PDXCs.

Supplementary Figure S9 CHK1 pharmacological inhibition mildly synergizes with Carboplatin in PDXC T-786

Supplementary Figure S10 Shift from γH2AX foci to pan-nuclear staining upon treatment with carboplatin and BAY1895344 in PDXC T-786

Supplementary Figure S11 A mitotic transcriptional program is triggered by the combination of carboplatin with ATRi in T-786 PDXC

Supplementary Figure S12 Transcriptomic modulation in response to treatment in PDXC T-786.

Supplementary Figure S13 RP-6306 shows different degrees of synergy with carboplatin in TNBC PDXCs

Supplementary Figure S14 WEE1 knockdown sensitizes PDXC T-786 to carboplatin.

Supplementary Figure S15 ATR and PKMYT1 double knockdown with siRNAs leads to an increase in the mitotic catastrophe induced by carboplatin.

Supplementary Figure S16 Molecular characteristics of models responding and not responding to Carbo-BAY combination

Supplementary Figure S17 Principal component analysis (PCA) shows a clear separation of responder and non-responder PDXs to the combination of carboplatin + ATRi.

Supplementary Methods Supplementary Methods

## Data Availability

The RNAseq data are available at Gene Expression Omnibus with the following accession number: GSE325338. All other data are available from the corresponding author upon request.
