# Supplementary material for: ATR and PKMYT1 Inhibition Resensitizes a Subset of TNBC Patient-Derived Models to Carboplatin, Inducing Mitotic Catastrophe
Source: Cancer Res Commun. 2026 May 12;6(5):1092–108. doi: 10.1158/2767-9764.CRC-25-0044 (PMC13161751; doi:10.1158/2767-9764.CRC-25-0044)
Supplement: Supplementary Table S1 — PDX origins [file crc-25-0044_supplementary_table_s1_suppst1.pdf]

Table S1. Patient-derived xenograft origin

Models	Origin	Treatment received*	Response to carboplatin
**PDX T-786**	Primary tumor	Car, T, A, C, Cape	Progressive disease
**PDX BM-156**	Metastasis	T, A, C	Progressive disease**
**PDX-1939**	Primary tumor	A, C, T, D	-
**PDX-1735**	Primary tumor	A, C, T, H	-
**BM-173**	Metastasis	T, A, C, Abr +/-At	-

*A, Doxorubicin; Abr, Abraxane; At, Atezolizumab; C, Cyclophosphamide; Cape, Capecitabine; Car, Carboplatin; D, Docetaxel; H, Trastuzumab; T, Paclitaxel

** BM-156 patient received carboplatin after the PDX was generated.
