# Supplementary material for: ATR and PKMYT1 Inhibition Resensitizes a Subset of TNBC Patient-Derived Models to Carboplatin, Inducing Mitotic Catastrophe
Source: Cancer Res Commun. 2026 May 12;6(5):1092–108. doi: 10.1158/2767-9764.CRC-25-0044 (PMC13161751; doi:10.1158/2767-9764.CRC-25-0044)
Supplement: Supplementary Table S2 — Drug concentrations [file crc-25-0044_supplementary_table_s2_suppst2.pdf]

Table 2. Drug concentrations associated with each assay

Drugs	Cell viability	Clonogenic assay	Western blot	Immunofluorescence	Annexin V	Cell cycle distribution	Distributor	Ref
**Carboplatin**	Gradient concentrations ranging from 500μM to 400nM<br>Fix concentrations of 500nM, 5μM, 10μM, 25μM	500nM	10μM, 20μM, 35μM	20μM	35μM	20μM	JGH, Dept Oncology Pharmacy	-
**Elimusertib**	Gradient concentrations ranging from 5μM to 2nM	5nM	5μM, 35μM	5nM	5nM	35nM	Selleckchem	S8666
**Ceralasertib**	Gradient concentrations ranging from 10μM to 40nM	20nM	200nM, 250nM, 1μM	250nM	200nM	250nM	Selleckchem	S7693
**Lunresertib**	Gradient concentrations ranging from 2.5μM to 10nM	-	-	-	-	-	Repare Therapeutics	-
**Adavosertib**	Gradient concentrations ranging from 2.5μM to 10nM	-	-	-	-	-	Selleckchem	S1525

Elimusertib, BAY1895344; Ceralasertib, AZD6738; Lunresertib, RP-6306; Adavosertib, AZD1775
