# Supplementary material for: ATR and PKMYT1 Inhibition Resensitizes a Subset of TNBC Patient-Derived Models to Carboplatin, Inducing Mitotic Catastrophe
Source: Cancer Res Commun. 2026 May 12;6(5):1092–108. doi: 10.1158/2767-9764.CRC-25-0044 (PMC13161751; doi:10.1158/2767-9764.CRC-25-0044)
Supplement: Supplementary Table S3 — shRNA, siRNA and primers sequences [file crc-25-0044_supplementary_table_s3_suppst3.pdf]

Table 3. List of shRNA, siRNA and primers sequences

Product	Target	ID	Provider	Sequence
**shRNA**	ATR	#1 (TRCN0000039615)	McGill Platform for Cellular Perturbation of the Goodman Cancer Research Centre	5'-CCGG-GCCGCTAATCTTCTAACATTA-CTCGAG-TAATGTTAGAAGATTAGCGGC-TTTTTG-3'
**shRNA**	ATR	#2 (TRCN0000039616)	McGill Platform for Cellular Perturbation of the Goodman Cancer Research Centre	5'-CCGG-GCCAAAGTATTTCTAGCCTAT-CTCGAG-ATAGGCTAGAAATACTTTGGC-TTTTTG-3'
**siRNA (pooled)**	PKMYT1	9088	Dharmacon	5’-GGACAGCAGCGGAUGUGUU-3’<br>5’-GGACCUAAGUGACAUCAAC-3’<br>5’-GAACCUCCUCAGCCUGUUU-3’<br>5’-CUUCCGAGCUGCGUUCUGU-3’
**siRNA (pooled)**	WEE1	7465	Dharmacon	5’-AAUAGAACAUCUCGACUUA-3’<br>5’-AAUAUGAAGUCCCGGUAUA-3’<br>5’-GAUCAUAUGCUUAUACAGA-3’<br>5’-CGACAGACUCCUCAAGUGA-3’
**siRNA**	ATR	82	ThermoFisher	5’-GGAAUAUAAUACAGUUGUATT-3’<br>5’-UACAACUGUAUUAUAUUCCTC-3’
**siRNA**	ATR	83	ThermoFisher	5’-GGCAAGAACUCUUACUUUGTT-3’<br>5’-CAAAGUAAGAGUUCUUGCCTT-3’
**siRNA**	ATR	s536	ThermoFisher	5’-UUGUAGAAAUGGAUACUGATT-3’<br>5’-UCAGUAUCCAUUUCUACAAGG-3’
**Primers**	Human PTGER2	-	-	Forward: 5’-GCTGCTTCTCATTGTCTCGG-3’<br>Reverse: 5’-GCCAGGAGAATGAGGTGGTC-3’
**Primers**	Mouse PTGER2	-	-	Forward: 5’-CCTGCTGCTTATCGTGGCTG-3’<br>Reverse: 5’-GCCAGGAGAATGAGGTGGTC-3’
**Primers**	ATR			Forward: 5’-CCGCAAAAGGAGATTTGGTA-3’<br>Reverse: 5’-TTCGGAAGTGCTGTCATCTG-3’
**Primers**	GAPDH			Forward: 5’-GGATTTGGTCGTATTGGG-3’<br>Reverse: 5’-GGAAGATGGTGATGGGATT-3’
