# Supplementary material for: ATR and PKMYT1 Inhibition Resensitizes a Subset of TNBC Patient-Derived Models to Carboplatin, Inducing Mitotic Catastrophe
Source: Cancer Res Commun. 2026 May 12;6(5):1092–108. doi: 10.1158/2767-9764.CRC-25-0044 (PMC13161751; doi:10.1158/2767-9764.CRC-25-0044)
Supplement: Supplementary Table S4 — List of antibodies [file crc-25-0044_supplementary_table_s4_suppst4.pdf]

Table S4. List of antibodies

Target	Dilution	Solution	Species	Distributor	Ref	RRID
**p-ATR T1989**	1:1000	TBST 5% BSA	Rabbit	Cell signaling	306325	AB_2798992
**ATR**	1:500	TBST 5% BSA	Rabbit	Cell signaling	13934	AB_2798347
**ATR**	1:500	TBST 5% BSA	Mouse	Santa cruz	515173	AB_2893291
**p-CHK1 S345**	1:1000	TBST 5% BSA	Rabbit	Cell signaling	2348	AB_331212
**CHK1**	1:1000	TBST 5% BSA	Mouse	Cell signaling	2360	AB_2080320
**p-ATM T1981**	1:1000	TBST 5% BSA	Rabbit	Cell signaling	13050	AB_2798100
**ATM**	1:1000	TBST 5% BSA	Mouse	Cell signaling	92356	AB_2800184
**γH2AX**	1:500	TBST 5% BSA	Rabbit	Millipore	JBW301	AB_309864
**H2AX**	1:1000	TBST 5% BSA	Mouse	Millipore	07-627	AB_2233033
**c-PARP**	1:1000	TBST 5% BSA	Mouse	Cell signaling	9546	AB_2160593
**p-HH3**	1:1000	TBST 5% Milk	Rabbit	Cell signaling	9701	AB_331535
**HH3**	1:1000	TBST 5% BSA	Rabbit	Cell signaling	4499	AB_10544537
**p-PKMYT1 S83**	1:1000	TBST 5% BSA	Rabbit	Cell signaling	4281	AB_2163832
**PKMYT1**	1:1000	TBST 5% BSA	Rabbit	Cell signaling	4281	AB_2163831
**p-CDK1 T14**	1:1000	TBST 5% Milk	Rabbit	Cell signaling	2543	AB_823465
**p-CDK1 Y15**	1:1000	TBST 5% Milk	Rabbit	Cell signaling	9111	AB_331460
**CDK1**	1:1000	TBST 5% Milk	Mouse	Invitrogen	MA5-11472	AB_10979734
**WEE1**	1:1000	TBST 5% BSA	Rabbit	Cell signaling	13084	AB_2713924
**p-FOXM1 T600**	1:1000	TBST 5% BSA	Rabbit	Cell signaling	14655	AB_2798557
**FOXM1**	1:1000	TBST 5% BSA	Rabbit	Cell signaling	20459	AB_2798842
**β-actin**	1:1000	TBST 5% BSA	Rabbit	Cell signaling	4967	AB_330288
**GAPDH**	1:1000	TBST 5% BSA	Rabbit	Cell signaling	2118	AB_561053
**HSP90**	1:1000	TBST 5% Milk	Rabbit	Cell signaling	8165	AB_11217436
**Tubulin**	1:1000	TBST 5% BSA	Mouse	Abcam	Ab7291	AB_2241126
**Anti-Rabbit HRP-linked**	1:10000	TBST	Goat	Cell signaling	7074	AB_2099233
**Anti-Mouse HRP-linked**	1:10000	TBST	Horse	Cell signaling	7076	AB_330924

TBST, Tris-buffered saline with 0.1% Tween 20 detergent; BSA, Bovine serum albumin
