# Supplementary material for: ATR and PKMYT1 Inhibition Resensitizes a Subset of TNBC Patient-Derived Models to Carboplatin, Inducing Mitotic Catastrophe
Source: Cancer Res Commun. 2026 May 12;6(5):1092–108. doi: 10.1158/2767-9764.CRC-25-0044 (PMC13161751; doi:10.1158/2767-9764.CRC-25-0044)
Supplement: Supplementary Table S5 — PDX responses to carboplatin and the carboATRi combination [file crc-25-0044_supplementary_table_s5_suppst5.pdf]

Table S5. PDXCs IC_{50} to carboplatin, BAY1895344 and AZD6738

PDXCs	Carboplatin IC_{50}	BAY IC_{50}	AZD IC_{50}
**T-786**	52μM	32.8nM	178.1nM
**T-817**	26μM	103.5nM	1.348μM
**T-830**	250μM	68.4nM	569.8nM
**BM-156**	3.5μM	78.28nM	579.8nM
