# Supplementary material for: ATR and PKMYT1 Inhibition Resensitizes a Subset of TNBC Patient-Derived Models to Carboplatin, Inducing Mitotic Catastrophe
Source: Cancer Res Commun. 2026 May 12;6(5):1092–108. doi: 10.1158/2767-9764.CRC-25-0044 (PMC13161751; doi:10.1158/2767-9764.CRC-25-0044)
Supplement: Supplementary Table S6 — Results of pooled shRNA ATR screen [file crc-25-0044_supplementary_table_s6_suppst6.pdf]

Table S6. Relative abundance of ATR-targeting shRNAs in the pooled screen under vehicle or carboplatin treatment, and the ratio of read counts (vehicle/carboplatin)

shRNA ID	Gene	Vehicle	Carboplatin	Ratio
**TRCN0000010300**	ATR	18	3	5.694
**TRCN0000010301**	ATR	57	0	n/a
**TRCN0000039613**	ATR	164	9	17.292
**TRCN0000039615**	ATR	213	32	6.738
**TRCN0000039616**	ATR	1871	352	5.316
**TRCN0000039614**	ATR	273	65	4.178
**TRCN0000039617**	ATR	4371	1569	2.786
**TRCN0000010302**	ATR	988	418	2.362
