# Supplementary material for: ATR and PKMYT1 Inhibition Resensitizes a Subset of TNBC Patient-Derived Models to Carboplatin, Inducing Mitotic Catastrophe
Source: Cancer Res Commun. 2026 May 12;6(5):1092–108. doi: 10.1158/2767-9764.CRC-25-0044 (PMC13161751; doi:10.1158/2767-9764.CRC-25-0044)
Supplement: Supplementary Table S7 — PDX responses to carboplatin and combination therapy [file crc-25-0044_supplementary_table_s7_suppst7.pdf]

Table S7. PDX responses to carboplatin and the combination carboplatin+ATRi summary

Models	Response to carboplatin	Eventual relapse with carboplatin	Response to combination	Eventual relapse with combination
**PDX T-786**	Resistant	-	Sensitive	-
**PDX BM-156**	Resistant	-	Sensitive	-
**PDX-1735**	Resistant	-	Sensitive	No
**PDX-1939**	Sensitive	Yes	Sensitive	No
**PDX BM-173**	Sensitive	Yes	Sensitive	2/4 mice relapsed
**PDX-1915**	Resistant	-	Resistant	-
**PDX-1924**	Resistant	-	Resistant	-
**PDX-1971**	Resistant	-	Resistant	-
**PDX-1986**	Resistant	-	Resistant	-
**PDX-2076**	Resistant	-	Resistant	-
**PDX-2089**	Resistant	-	Resistant	-
**PDX-1886**	Sensitive	No	Sensitive	No
**PDX-1905**	Sensitive	Yes	Sensitive	Yes
**PDX-1945**	Sensitive	No	Sensitive	No
