# Supplementary material for: ATR and PKMYT1 Inhibition Resensitizes a Subset of TNBC Patient-Derived Models to Carboplatin, Inducing Mitotic Catastrophe
Source: Cancer Res Commun. 2026 May 12;6(5):1092–108. doi: 10.1158/2767-9764.CRC-25-0044 (PMC13161751; doi:10.1158/2767-9764.CRC-25-0044)
Supplement: Supplementary Table S8 — Differentially expressed genes in response to carboplatin [file crc-25-0044_supplementary_table_s8_suppst8.pdf]

Table S8. Differentially expressed genes in response to carboplatin

Genes	T-786_UT (average read)	T-786_CARBO (average read)	log2FoldChange	padj_value	CARBO + BAY log2FoldChange p<0.05	CARBO + RP log2FoldChange p<0.05
CLSPN	1833	2331	0.39231	0.00021	-0.55703	-0.29252
EXO1	1637	2090	0.39422	0.00147	-0.61912	-0.25034
BLM	1787	2163	0.32258	0.02744	-0.42577	-
CDC6	2232	2738	0.34349	0.03737	-0.52031	-
ORC1	698	861	0.35139	0.04610	-0.41717	-
E2F2	354	458	0.42092	0.04812	-0.63515	-
KIF20A	3025	1896	-0.63098	3.02E-10	1.16811	0.46510
DEPDC1	2208	1425	-0.58792	1.10E-08	0.96485	0.30336
GAS2L3	933	552	-0.70981	1.23E-08	0.75806	0.32227
PLK1	1981	1236	-0.63675	4.71E-07	1.06947	0.64028
CCNB1	14699	9746	-0.54986	1.44E-06	1.02500	0.54679
TPX2	7788	5839	-0.36839	8.71E-06	0.47573	0.27382
CDC20	3115	2236	-0.43034	2.68E-05	1.04798	0.49305
FAM83D	1320	910	-0.49363	6.35E-05	0.55147	0.33049
ASPM	6534	4873	-0.37931	0.00017	0.52090	-
H1F0	203	100	-0.95789	0.00018	1.22580	0.97812
DLGAP5	2411	1787	-0.38480	0.00025	0.70877	-
KIF23	5111	3852	-0.36137	0.00025	0.57582	-
AURKA	2686	1901	-0.45695	0.00087	0.76619	0.37110
KIF14	2293	1705	-0.38372	0.00087	0.68414	0.25310
CENPE	4083	2862	-0.47584	0.00105	0.67514	0.31418
CENPA	472	294	-0.64510	0.00122	0.83278	0.62987
SGOL2	1009	721	-0.44132	0.00142	0.76889	-
CENPF	10963	8190	-0.38214	0.00257	0.62181	-
NCAPD2	17362	13544	-0.31369	0.00433	0.59752	0.24430
PIF1	173	94	-0.83252	0.00758	1.30615	0.79578
GTSE1	1115	816	-0.39933	0.00767	0.71338	-
ANLN	7076	5480	-0.32230	0.00922	0.36840	-
PSRC1	207	116	-0.78761	0.01045	1.00466	0.62661
CKAP2	2568	2015	-0.30129	0.01105	0.49686	-
SUN2	702	493	-0.45728	0.01275	0.79258	0.38233
KIF18A	919	678	-0.38912	0.01275	0.48935	0.32546
RACGAP1	2873	2244	-0.30891	0.01351	0.46205	0.22632
DEPDC1B	891	659	-0.39023	0.01386	0.31349	-
TNFAIP8L1	165	89	-0.85773	0.01767	0.75929	-
CDCA8	961	727	-0.35422	0.02337	0.62325	0.41738
HMMR	3731	2886	-0.32534	0.02337	0.64900	-
TOP2A	15649	12440	-0.28826	0.02512	0.59031	-
CCNB2	3184	2427	-0.34797	0.03623	0.74672	0.34153
NDC80	2483	1985	-0.27650	0.03965	0.33836	-
ECT2	5903	4741	-0.26847	0.03974	0.40611	-
G2E3	1199	908	-0.35084	0.04048	0.42673	-
HIST1H2AC	513	333	-0.56384	0.04670	1.25878	0.74041
PTTG1	1441	1042	-0.42668	0.04670	0.90812	0.53299
TRIM59	389	269	-0.49089	0.04721	0.68330	0.39324
