# Supplementary material for: ATR and PKMYT1 Inhibition Resensitizes a Subset of TNBC Patient-Derived Models to Carboplatin, Inducing Mitotic Catastrophe
Source: Cancer Res Commun. 2026 May 12;6(5):1092–108. doi: 10.1158/2767-9764.CRC-25-0044 (PMC13161751; doi:10.1158/2767-9764.CRC-25-0044)
Supplement: Supplementary Figure S1 — Genomic Fidelity of PDX models [file crc-25-0044_supplementary_figure_s1_suppsf1.pdf]

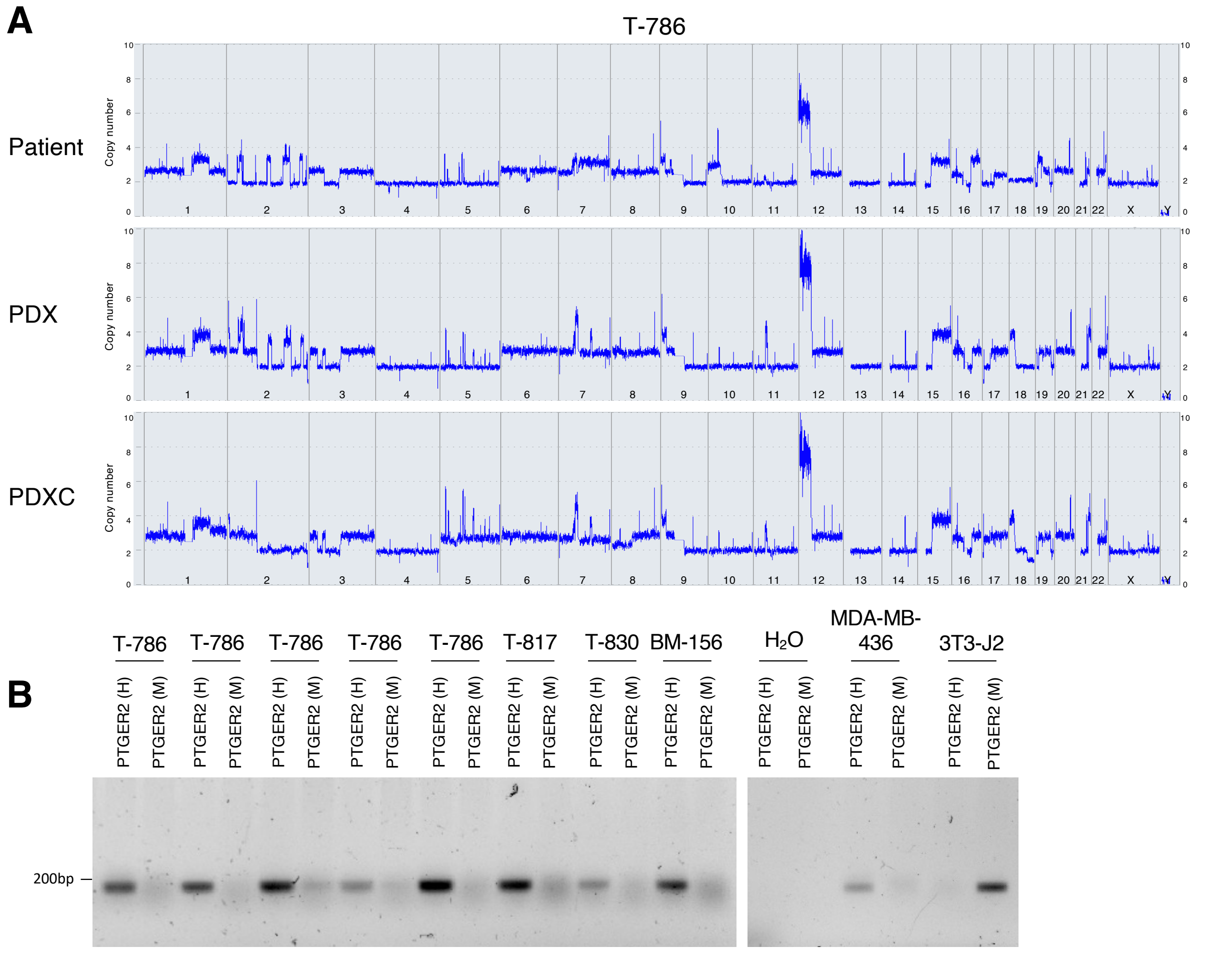

Supplementary Figure S1: PDXCs generated from the PDXs show a high degree of genomic fidelity with the PDX and the patient tumor of origin and are exempt of mouse cells.

A. Whole genome view of copy number alterations in T-786 patient tumor (top), associated patient-derived xenograft (PDX), and associated patient-derived xenograft cells (PDXC) generated with Chromosome Analysis Suite (ChAS 3.1). The blue line represents the smooth signal of copy number (Y axis) for all the chromosomes (one chromosome per panel) **B**. End point PCR results confirming the human origin of each PDXC. PCR Amplification products were loaded and migrated on agarose gel. Presence of human cells (amplification product with the human (H) PTGER2 primers) and absence of mouse cells (amplification product with the mouse (M) PTGER2 primers) is seen in PDXC T-786 (several passages shown), PDXC T-817, PDXC T-830, PDXC BM-156. MDA-MB-436 and 3T3-J2 cells were used respectively as human and mouse cells control.
