# Supplementary material for: ATR and PKMYT1 Inhibition Resensitizes a Subset of TNBC Patient-Derived Models to Carboplatin, Inducing Mitotic Catastrophe
Source: Cancer Res Commun. 2026 May 12;6(5):1092–108. doi: 10.1158/2767-9764.CRC-25-0044 (PMC13161751; doi:10.1158/2767-9764.CRC-25-0044)
Supplement: Supplementary Figure S2 — Carboplatin resistant PDXs [file crc-25-0044_supplementary_figure_s2_suppsf2.pdf]

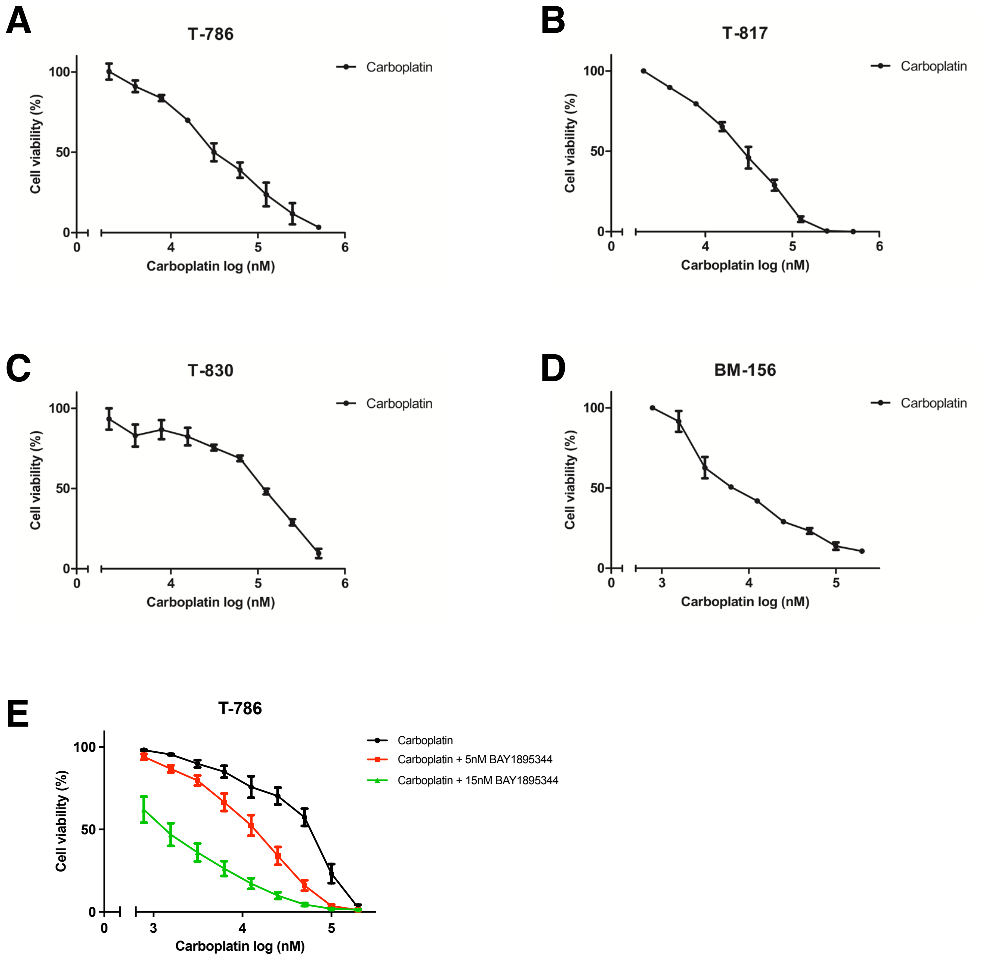

Supplementary Figure S2: Carboplatin-resistant TNBC PDXCs

A. Cell viability assay (%) in response to carboplatin was determined using the Alamar Blue in PDXC T-786, **B**. PDXC T-817, **C**. and PDXC T-830. **D**. and Sulforhodamine B assay in PDXC BM-156. **E**. Cell viability assay in response to carboplatin with 5nM or 15nM of BAY1895344 was determined using the Alamar Blue in PDXC T-786.
