# Supplementary material for: ATR and PKMYT1 Inhibition Resensitizes a Subset of TNBC Patient-Derived Models to Carboplatin, Inducing Mitotic Catastrophe
Source: Cancer Res Commun. 2026 May 12;6(5):1092–108. doi: 10.1158/2767-9764.CRC-25-0044 (PMC13161751; doi:10.1158/2767-9764.CRC-25-0044)
Supplement: Supplementary Figure S3 — ATR knockdown impairs proliferation of PDXC T-786 cells. [file crc-25-0044_supplementary_figure_s3_suppsf3.pdf]

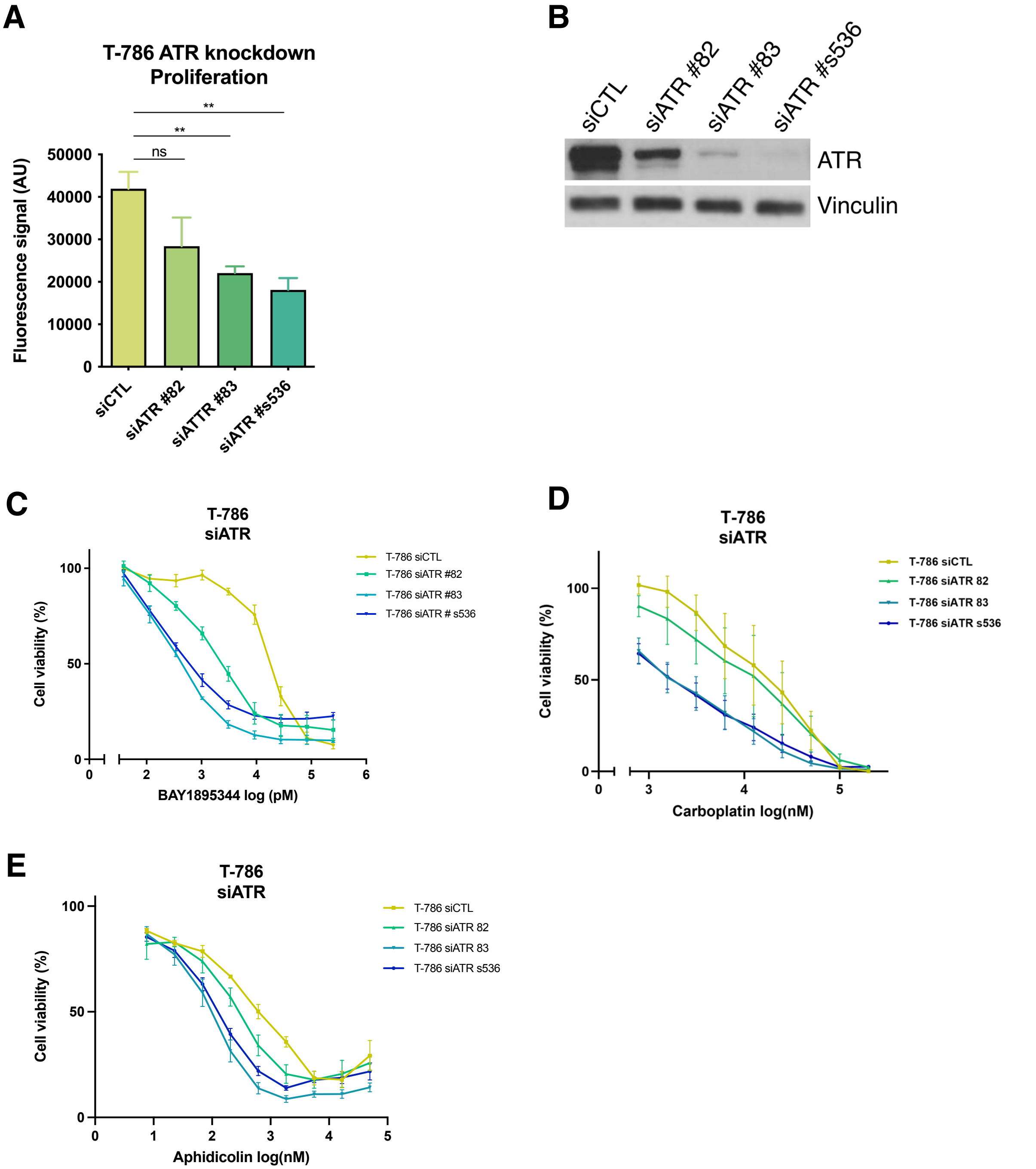

Supplementary Figure S3: ATR knockdown impairs proliferation of PDXC T-786 cells.

A..**B.** Effect of transient ATR knockdown using three independent siRNAs (#82, #83, and #s536) on the proliferation of PDXC T-786. Cells were transfected for 24 hours, seeded, and assessed for proliferation using Alamar Blue assay after 3 days. Fluorescence intensity reflects relative cell viability, **P < 0.01, n=3. **C**. Cell viability assay of ATR knockdown PDXC T-786 cells exposed to a gradient concentration of BAY1895344, n=3. **D**. Cell viability assay of ATR knockdown PDXC T-786 cells exposed to a gradient concentration of aphidicolin, n=3. **E.** Cell viability assay of ATR knockdown PDXC T-786 cells exposed to a gradient concentration of carboplatin, n=3. **F.** Immunoblot showing the validation of ATR knockdown using three independent siRNAs (#82, #83, and #s536).
