# Supplementary material for: ATR and PKMYT1 Inhibition Resensitizes a Subset of TNBC Patient-Derived Models to Carboplatin, Inducing Mitotic Catastrophe
Source: Cancer Res Commun. 2026 May 12;6(5):1092–108. doi: 10.1158/2767-9764.CRC-25-0044 (PMC13161751; doi:10.1158/2767-9764.CRC-25-0044)
Supplement: Supplementary Figure S4 — CDK2 silencing results [file crc-25-0044_supplementary_figure_s4_suppsf4.pdf]

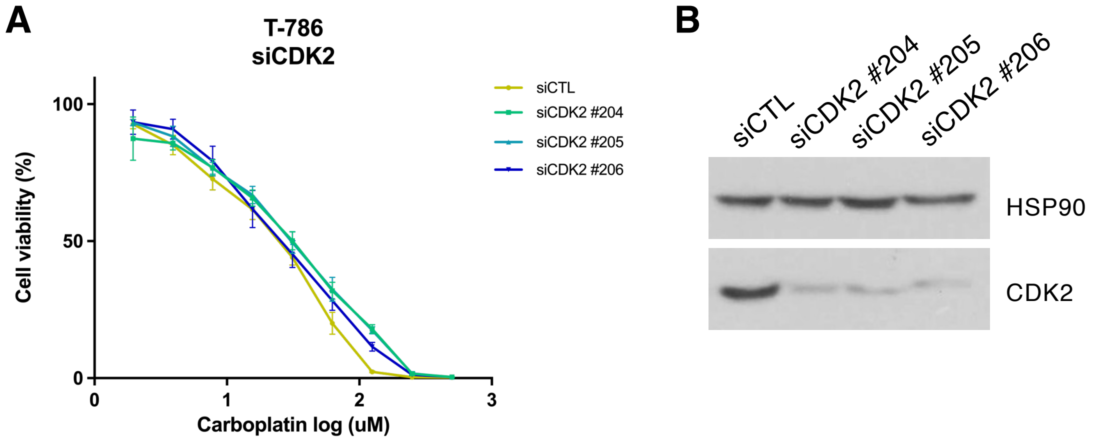

Supplementary Figure S4: CDK2 silencing did not re-sensitize PDXC T-786 to carboplatin.
A. Cell viability assay of CDK2 knockdown PDXC T-786 cells exposed to a gradient concentration of carboplatin, n=3 .**B.** Immunoblot showing the validation of CDK2 knockdown using three independent siRNAs (#204, #205, and #206).
