# Supplementary material for: ATR and PKMYT1 Inhibition Resensitizes a Subset of TNBC Patient-Derived Models to Carboplatin, Inducing Mitotic Catastrophe
Source: Cancer Res Commun. 2026 May 12;6(5):1092–108. doi: 10.1158/2767-9764.CRC-25-0044 (PMC13161751; doi:10.1158/2767-9764.CRC-25-0044)
Supplement: Supplementary Figure S5 — Combination AZD6738 and carboplatin [file crc-25-0044_supplementary_figure_s5_suppsf5.pdf]

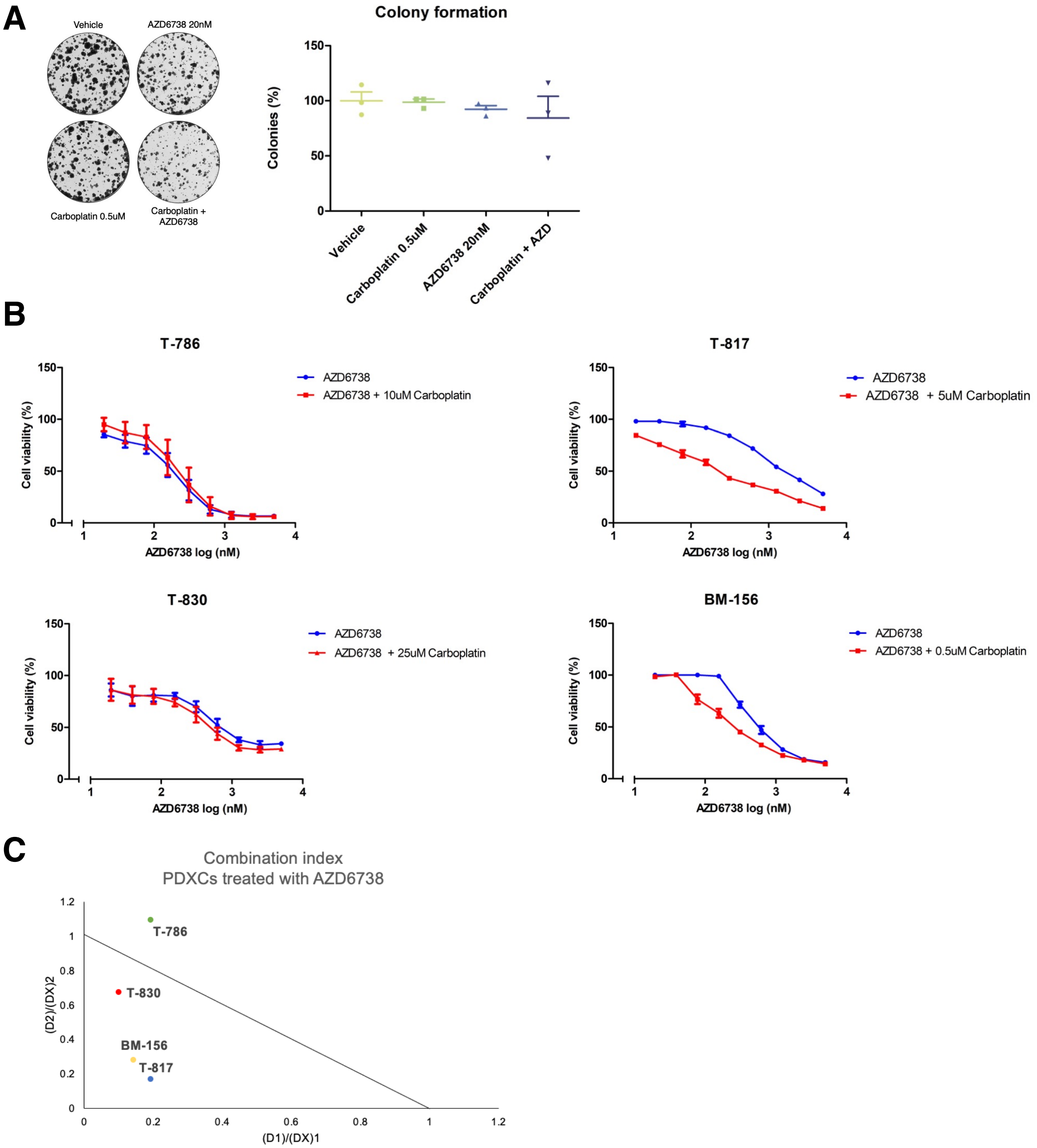

Supplementary Figure S5: Pharmacological inhibition of ATR with AZD6738 synergizes with carboplatin in selected TNBC PDXCs.

A. Representative images of clonogenic assay of PDXC T-786 treated for 14 days with vehicle, 20nM AZD6738 alone, 0.5μM carboplatin alone, and 20nM AZD6738 alone + 0.5μM Carboplatin (left), and the quantification (right). **B**. Cell viability (%) of TNBC PDXCs treated with the indicated drug combinations and measured by AlamarBlue assay. IC_{10} to IC_{25} concentrations of carboplatin were used in combination to a gradient concentration AZD6738 (n=3). **C**. Isobolograms representing the combination indexes of each TNBC PDXCs for AZD6738 combined with carboplatin.
