# Supplementary material for: ATR and PKMYT1 Inhibition Resensitizes a Subset of TNBC Patient-Derived Models to Carboplatin, Inducing Mitotic Catastrophe
Source: Cancer Res Commun. 2026 May 12;6(5):1092–108. doi: 10.1158/2767-9764.CRC-25-0044 (PMC13161751; doi:10.1158/2767-9764.CRC-25-0044)
Supplement: Supplementary Figure S6 — Toxicity of ATR inh and carboplatin [file crc-25-0044_supplementary_figure_s6_suppsf6.pdf]

A

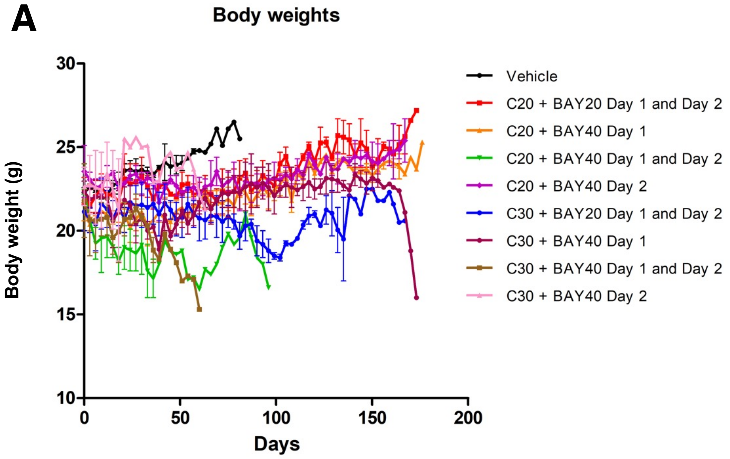

Supplementary Figure S6: Carboplatin-BAY1895344 combination tolerability in NSG mice and efficacy in PDX T_786 and BM-156.

A. Body weight in response to different schedules and combinations of carboplatin 20mg/kg (C20) or carboplatin 30mg/kg (C30) and BAY1895344 20mg/kg (BAY20) or and BAY1895344 40mg/kg (BAY40). Carboplatin was administered once a week, and BAY was administered either once (Day 1 or Day 2) or twice (Day 1 and Day 2) a week.
