# Supplementary material for: ATR and PKMYT1 Inhibition Resensitizes a Subset of TNBC Patient-Derived Models to Carboplatin, Inducing Mitotic Catastrophe
Source: Cancer Res Commun. 2026 May 12;6(5):1092–108. doi: 10.1158/2767-9764.CRC-25-0044 (PMC13161751; doi:10.1158/2767-9764.CRC-25-0044)
Supplement: Supplementary Figure S7 — TNBC PDX small trials [file crc-25-0044_supplementary_figure_s7_suppsf7.pdf]

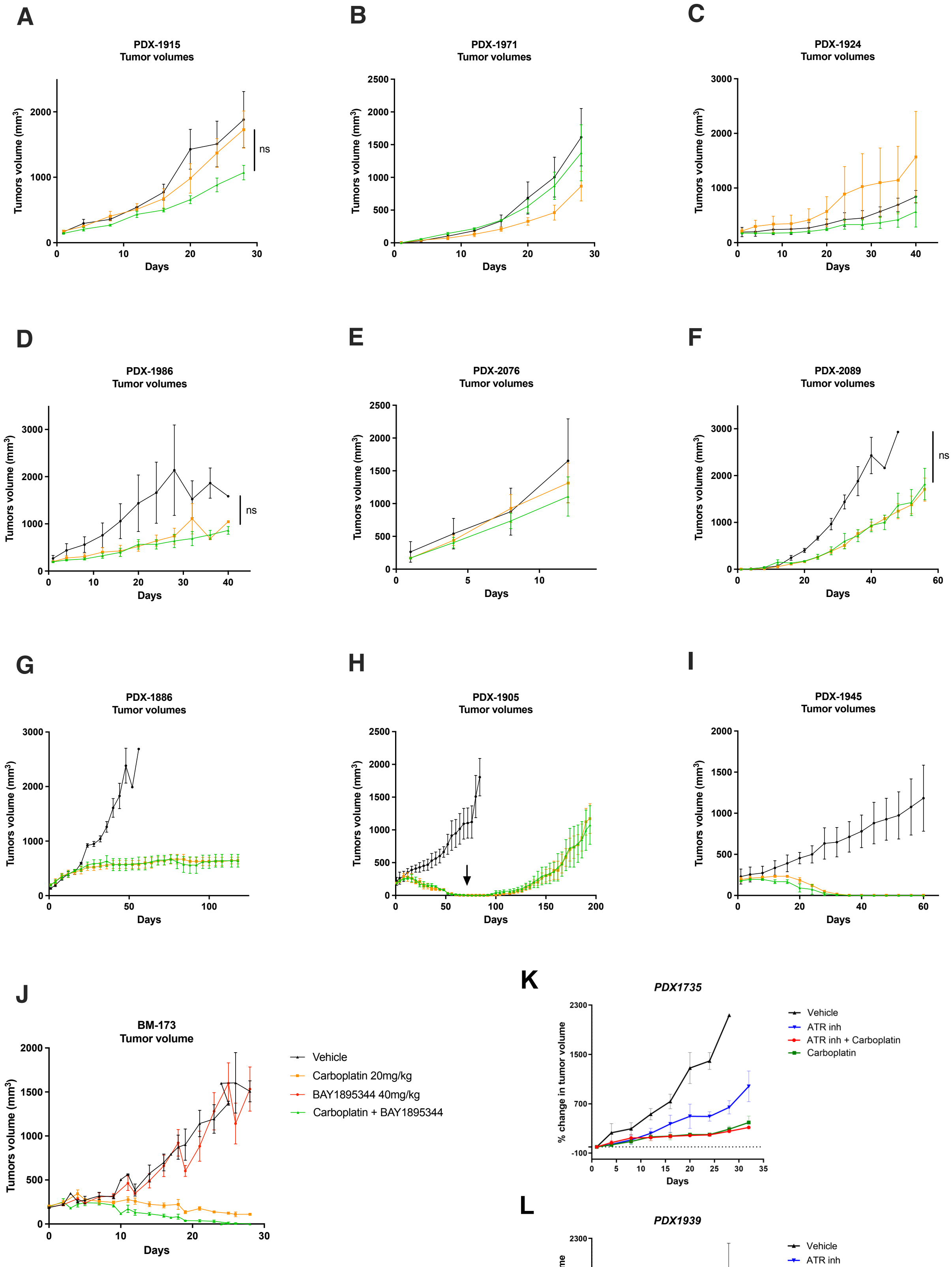

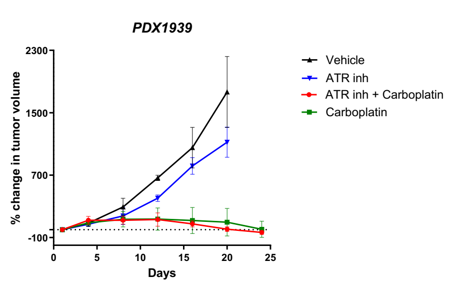

Supplementary Figure S7: TNBC PDXs small trials (n=3)
Tumor growth of TNBC PDXs treated with vehicle (black) (n=3), carboplatin (red) 20mg/kg (n=3) or carboplatin 20mg/kg + BAY1895344 (green) 40mg/kg (n=3): Carboplatin resistant models: **A**. PDX-1915, **B**. PDX-1971, **C**. PDX-1924, **D**. PDX-1986, **E**. PDX-2076, **F**. PDX-2089. Or carboplatin-sensitive models: **G**. PDX-1886 and **H**. PDX-1905. **I.** PDX-1945. Validation study to assess the effect of BAY1895344 alone in **J.** PDX BM-173, **K**. PDX-1735 and **L**. PDX-1939. Each treatment arm comprises 5 to 6 mice.
