# Supplementary material for: ATR and PKMYT1 Inhibition Resensitizes a Subset of TNBC Patient-Derived Models to Carboplatin, Inducing Mitotic Catastrophe
Source: Cancer Res Commun. 2026 May 12;6(5):1092–108. doi: 10.1158/2767-9764.CRC-25-0044 (PMC13161751; doi:10.1158/2767-9764.CRC-25-0044)
Supplement: Supplementary Figure S8 — Effect of ATR inhibition on ATM-Chk2 pathway and ATR protein/mRNA levels in TNBC PDXCs. [file crc-25-0044_supplementary_figure_s8_suppsf8.pdf]

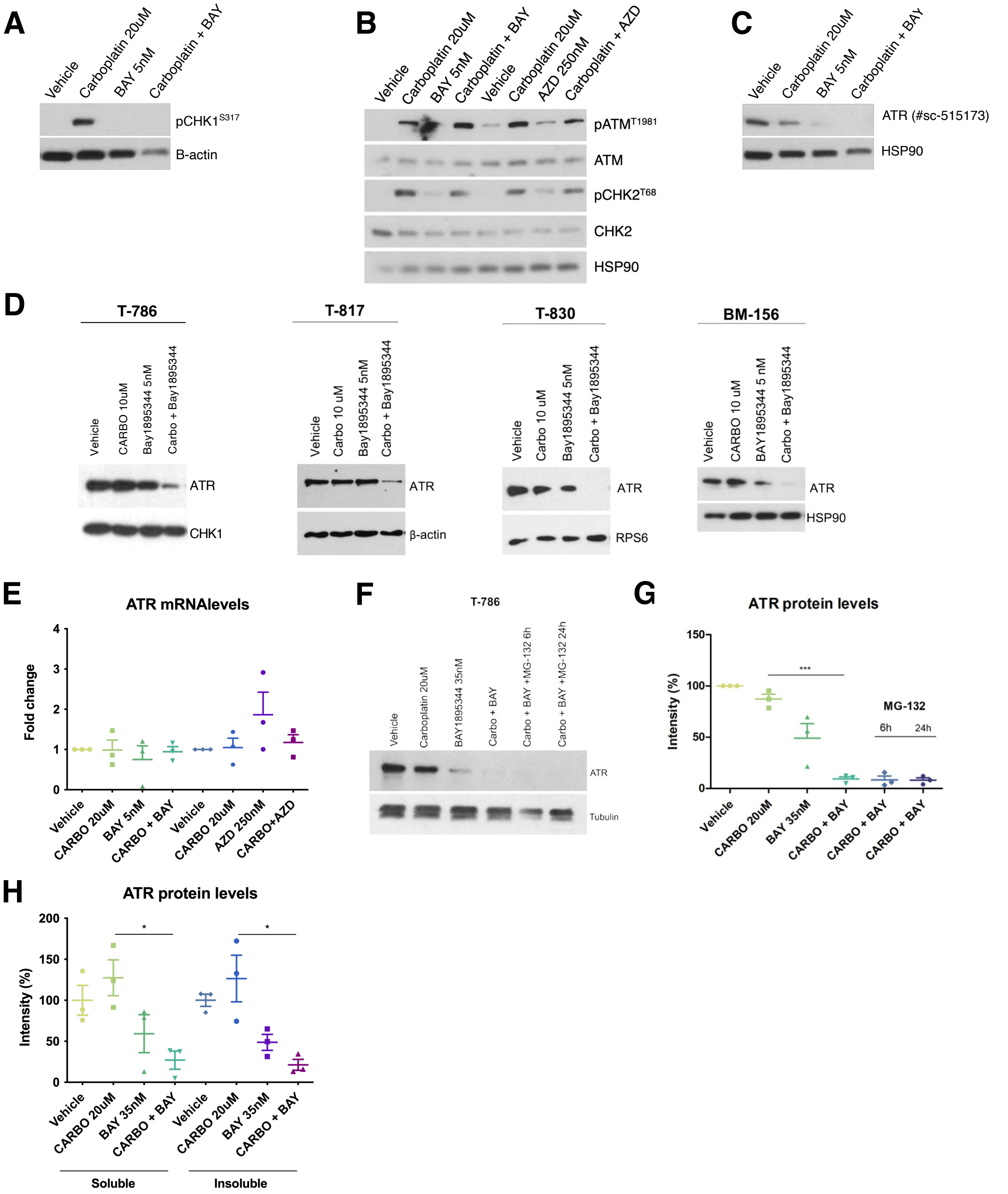

Supplementary Figure S8: Effect of ATR inhibition on ATM-Chk2 pathway and ATR protein/mRNA levels in TNBC PDXCs.

A. Immunoblot analysis Immunoblot analysis of CHK1 phosphorylation on S317 in response to vehicle (carbomethylcellulose 0.5%), 20µM carboplatin, 5nM BAY1895344 or carboplatin + BAY1895344 in PDXCs T-786. **B**. Immunoblot analysis of ATM (p-T1981) and Chk2 (T68) activation in response to the ATR inhibitors alone or in combination with carboplatin in T-786 PDXC. **C**. Immunoblot analysis Immunoblot analysis of ATR protein levels in response to vehicle, 20µM carboplatin, 5nM BAY1895344 or carboplatin + BAY1895344 in PDXCs T-786 using another ATR antibody: santa cruz #515173. **D.** Immunoblot analysis of ATR protein levels in response to vehicle, 10µM carboplatin, 5nM BAY1895344 or carboplatin + BAY1895344 in PDXCs T-786, T-817, T-830. and BM-156. **E**. Quantification of ATR mRNA levels by qPCR in response to vehicle, 20µM carboplatin, 5nM BAY1895344 or carboplatin + BAY1895344, vehicle (DMSO), 250nM AZD6738 or carboplatin + AZD6738 in PDXC (n=3). **F**. Immunoblot analysis of the effect of the addition of 10µM MG-132 to the carboplatin + BAYER1895344 combination for 6h or for the whole 24h of treatment on ATR protein levels. **G.** ATR protein levels quantification (n=3). **H**. Quantification of ATR protein levels in soluble and insoluble fractions of a fractionation assay. Significance assessed by the two-sided unpaired nonparametric Student's t test. Mean ± SEM, n=3, *$P < 0.05$, ***$P < 0.001$. Quantifications were calculated using ImageJ software. Region of interest (ROI) was determined for each lane where the intensity of the band was measured. The band intensity of the protein of interest was normalized on the band intensity of the loading control for each lane.
