# Supplementary material for: ATR and PKMYT1 Inhibition Resensitizes a Subset of TNBC Patient-Derived Models to Carboplatin, Inducing Mitotic Catastrophe
Source: Cancer Res Commun. 2026 May 12;6(5):1092–108. doi: 10.1158/2767-9764.CRC-25-0044 (PMC13161751; doi:10.1158/2767-9764.CRC-25-0044)
Supplement: Supplementary Figure S9 — CHK1 pharmacological inhibition mildly synergizes with Carboplatin in PDXC T-786 [file crc-25-0044_supplementary_figure_s9_suppsf9.pdf]

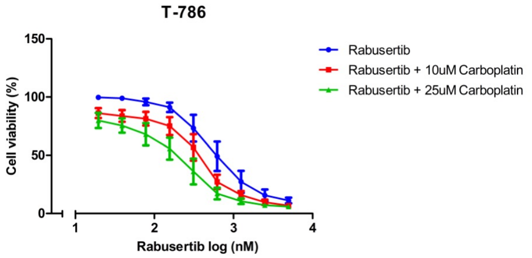

Supplementary Figure S9: CHK1 pharmacological inhibition mildly synergizes with Carboplatin in PDXC T-786
Cell viability (%) assay using Alamar blue of PDXC T-786 treated with a gradient concentration of Rabusertib (CHK1 inhibitor) alone, combined with 10μM carboplatin or 25μM carboplatin
