# Supplementary material for: ATR and PKMYT1 Inhibition Resensitizes a Subset of TNBC Patient-Derived Models to Carboplatin, Inducing Mitotic Catastrophe
Source: Cancer Res Commun. 2026 May 12;6(5):1092–108. doi: 10.1158/2767-9764.CRC-25-0044 (PMC13161751; doi:10.1158/2767-9764.CRC-25-0044)
Supplement: Supplementary Figure S10 — Shift from γH2AX foci to pan-nuclear staining upon treatment with carboplatin and BAY1895344 in PDXC T-786 [file crc-25-0044_supplementary_figure_s10_suppsf10.pdf]

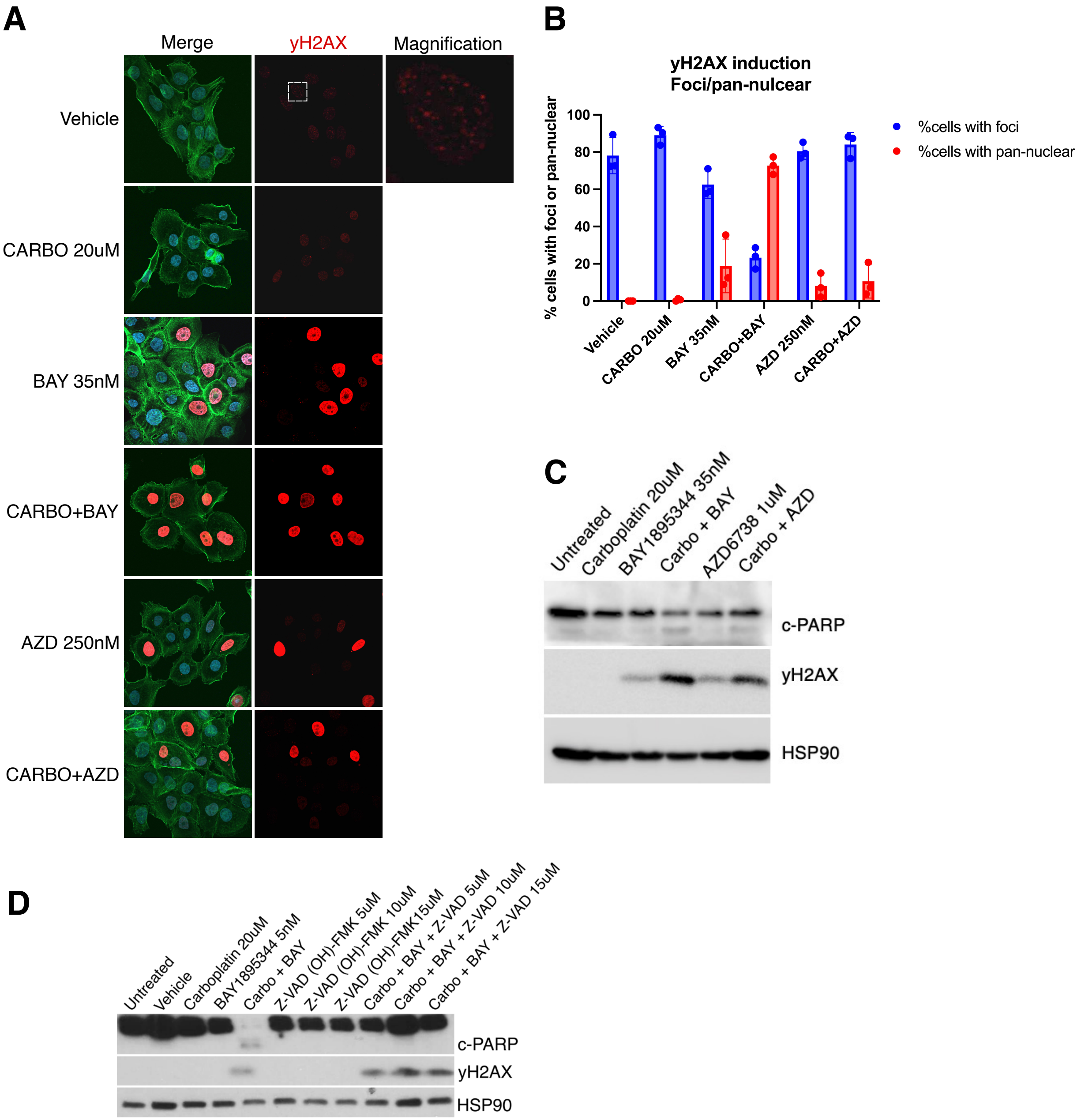

Supplementary Figure S10: Shift from γH2AX foci to pan-nuclear staining upon treatment with carboplatin and BAY1895344 in PDXC T-786
A. Representative images of immunofluorescence of γH2AX levels acquired by confocal microscopy in response to 48h of treatments. 40X magnification, γH2AX-Alexa fluor 594 (red), nucleus (blue), F-actin (green) **B.** Quantification of T-786 PDXC cells with γH2AX foci and pan-nuclear phenotype, n=3. **C**. Immunoblot analysis of apoptosis (cleaved-PARP) and DNA damage (γH2AX) in PDXC T-786 untreated or treated with the indicated drug concentrations for 48h. **D**. Immunoblot analysis of apoptosis (cleaved-PARP) and DNA damage (γH2AX) in PDXC T-786 treated with the indicated drug concentrations for 48h with of without indicated concentration of a pan-caspase inhibitor (Z-VAD(OH)-FMK)
