# Supplementary material for: ATR and PKMYT1 Inhibition Resensitizes a Subset of TNBC Patient-Derived Models to Carboplatin, Inducing Mitotic Catastrophe
Source: Cancer Res Commun. 2026 May 12;6(5):1092–108. doi: 10.1158/2767-9764.CRC-25-0044 (PMC13161751; doi:10.1158/2767-9764.CRC-25-0044)
Supplement: Supplementary Figure S11 — A mitotic transcriptional program is triggered by the combination of carboplatin with ATRi in T-786 PDXC [file crc-25-0044_supplementary_figure_s11_suppsf11.pdf]

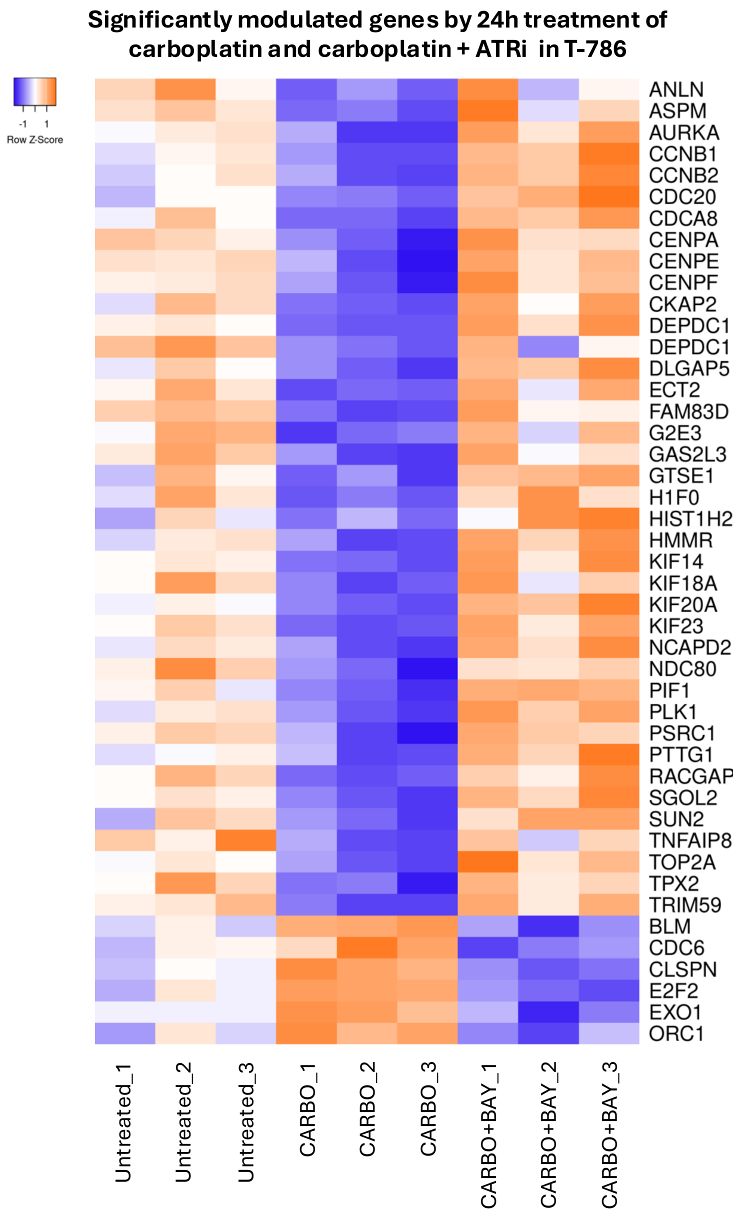

Supplementary Figure S11: A mitotic transcriptional program is triggered by the combination of carboplatin with ATRi in T-786 PDXC
Heatmap representing the significantly modulated genes by 24h treatment of carboplatin and carboplatin + ATRi in T-786 PDXC. Samples were processed in quadruplicates and represented genes were selected with P < 0.05.
