# Supplementary material for: ATR and PKMYT1 Inhibition Resensitizes a Subset of TNBC Patient-Derived Models to Carboplatin, Inducing Mitotic Catastrophe
Source: Cancer Res Commun. 2026 May 12;6(5):1092–108. doi: 10.1158/2767-9764.CRC-25-0044 (PMC13161751; doi:10.1158/2767-9764.CRC-25-0044)
Supplement: Supplementary Figure S12 — Transcriptomic modulation in response to treatment in PDXC T-786. [file crc-25-0044_supplementary_figure_s12_suppsf12.pdf]

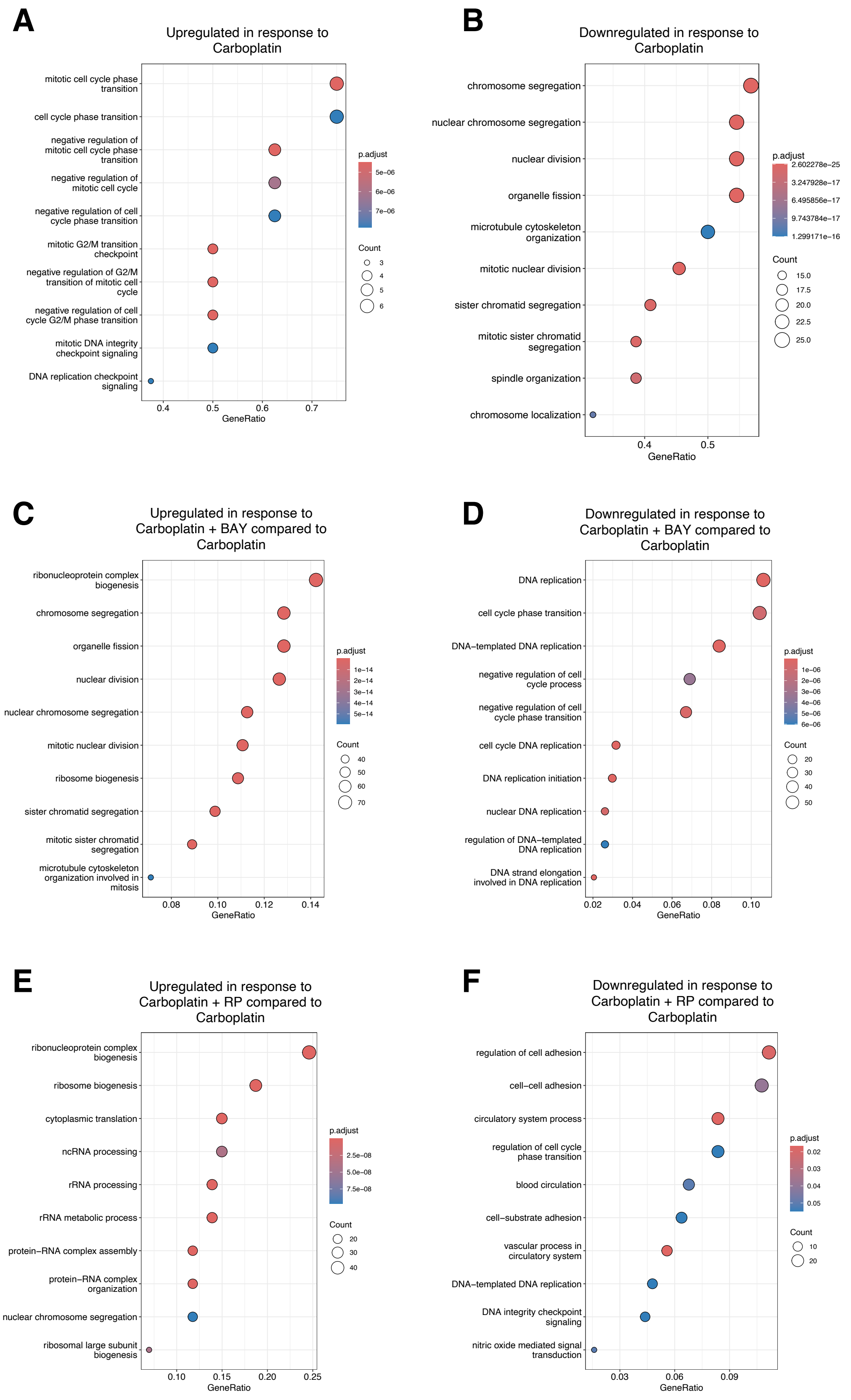

Supplementary Figure S12: Transcriptomic modulation in response to treatment in PDXC T-786

A. Dot plot showing the top 10 gene ontology (GO) biological processes enriched and **B**. suppressed in response to carboplatin compared to untreated PDXC T-786. **C**. Dot plot showing the top 10 gene ontology (GO) biological processes enriched and **D**. suppressed in response to carboplatin + BAY compared to carboplatin alone treated PDXC T-786. **E**. Dot plot showing the top 10 gene ontology (GO) biological processes enriched and **F**. suppressed in response to carboplatin + RP-6306 compared to carboplatin alone treated PDXC T-786. Processes were selected with a *p* adjusted value cut off 0.05.
