# Supplementary material for: ATR and PKMYT1 Inhibition Resensitizes a Subset of TNBC Patient-Derived Models to Carboplatin, Inducing Mitotic Catastrophe
Source: Cancer Res Commun. 2026 May 12;6(5):1092–108. doi: 10.1158/2767-9764.CRC-25-0044 (PMC13161751; doi:10.1158/2767-9764.CRC-25-0044)
Supplement: Supplementary Figure S13 — RP-6306 shows different degrees of synergy with carboplatin in TNBC PDXCs [file crc-25-0044_supplementary_figure_s13_suppsf13.pdf]

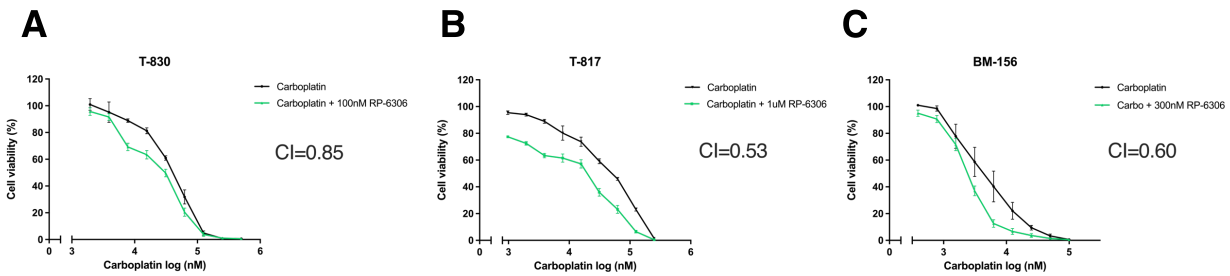

Supplementary Figure S13: RP-6306 shows different degrees of synergy with carboplatin in TNBC PDXCs.

A. Cell viability (%) of PDXCs T-830 (**A**), T-817 (**B**) and BM-156 (**C**) measured by Alamar blue assay in response to a concentration gradient of carboplatin combined with RP-6306 at concentrations corresponding to their respective IC25. (n=3). The Combination index (CI) is indicated for each cell line.
