# Supplementary material for: ATR and PKMYT1 Inhibition Resensitizes a Subset of TNBC Patient-Derived Models to Carboplatin, Inducing Mitotic Catastrophe
Source: Cancer Res Commun. 2026 May 12;6(5):1092–108. doi: 10.1158/2767-9764.CRC-25-0044 (PMC13161751; doi:10.1158/2767-9764.CRC-25-0044)
Supplement: Supplementary Figure S14 — WEE1 knockdown sensitizes PDXC T-786 to carboplatin. [file crc-25-0044_supplementary_figure_s14_suppsf14.pdf]

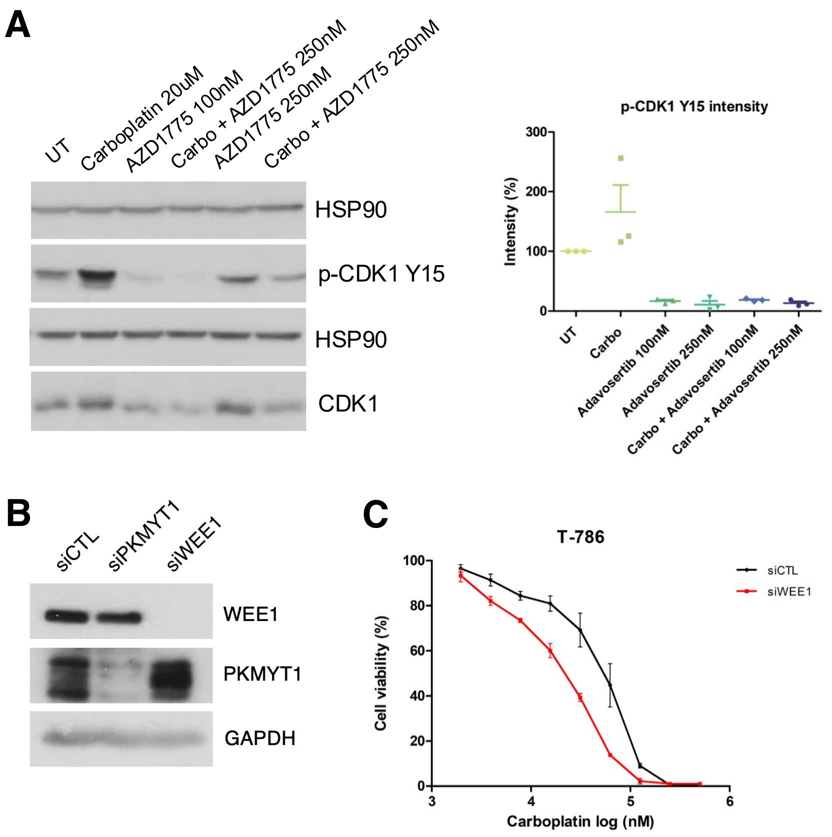

Supplementary Figure S14: WEE1 knockdown sensitizes PDXC T-786 to carboplatin.

A. Immunoblot analysis showing the effect of the WEE1 inhibitor AZD1775, alone or in combination with carboplatin, on CDK1 phosphorylation (Y15) in PDXC T-786 treated with the indicated drug combination. Quantification of CDK1 phosphorylation on Y15 is shown on the right (n=3). **B**. Immunoblot analysis of PKMYT1 and WEE1 levels in PDXC T-786 transfected with their respective pool of siRNA. **C.** Cell viability (%) of PDXC T-786 transfected with a pool of WEE1 siRNAs and treated with a gradient of carboplatin
