# Supplementary material for: ATR and PKMYT1 Inhibition Resensitizes a Subset of TNBC Patient-Derived Models to Carboplatin, Inducing Mitotic Catastrophe
Source: Cancer Res Commun. 2026 May 12;6(5):1092–108. doi: 10.1158/2767-9764.CRC-25-0044 (PMC13161751; doi:10.1158/2767-9764.CRC-25-0044)
Supplement: Supplementary Figure S15 — ATR and PKMYT1 double knockdown with siRNAs leads to an increase in the mitotic catastrophe induced by carboplatin. [file crc-25-0044_supplementary_figure_s15_suppsf15.pdf]

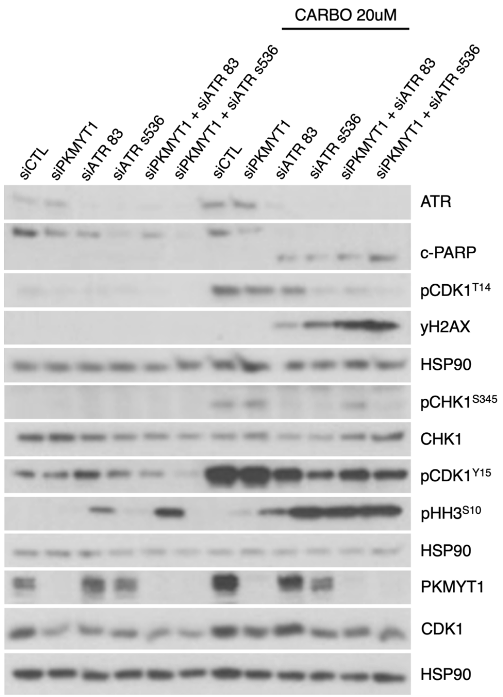

Supplementary Figure S15: ATR and PKMYT1 double knockdown with siRNAs leads to an increase in the mitotic catastrophe induced by carboplatin. Immunoblot analysis of mitosis (pHH3, CDK1/pCDK1, PKMYT1, CHK1/pCHK1), DNA damage (γH2AX) and apoptosis (c-PARP) markers in PDXC T-786 treated with ATR and/or PKMYT1 siRNAs and exposed of not to 20μM of carboplatin.
