# Supplementary material for: ATR and PKMYT1 Inhibition Resensitizes a Subset of TNBC Patient-Derived Models to Carboplatin, Inducing Mitotic Catastrophe
Source: Cancer Res Commun. 2026 May 12;6(5):1092–108. doi: 10.1158/2767-9764.CRC-25-0044 (PMC13161751; doi:10.1158/2767-9764.CRC-25-0044)
Supplement: Supplementary Figure S16 — Molecular characteristics of models responding and not responding to Carbo-BAY combination [file crc-25-0044_supplementary_figure_s16_suppsf16.pdf]

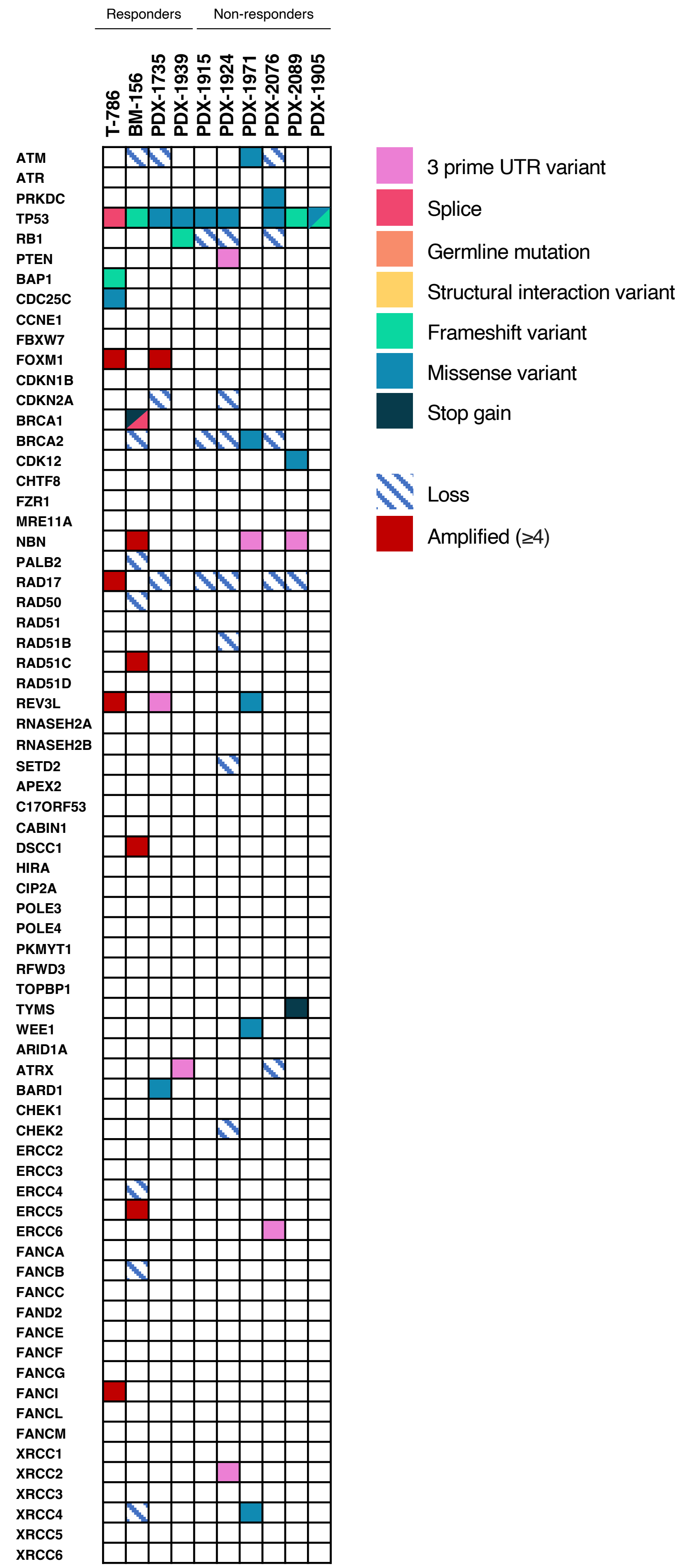

Supplementary Figure S16: Molecular characteristics of models responding and not responding to Carbo-BAY combination. Representation of gene alterations observed for most of the models used in this study. Genes selected based on the predictive value of response to ATR inhibitors based on the literature, general role in DNA damage or cell cycle, or commonly altered in cancer. Additional genes that were investigated that presented no alterations in none of the models were excluded. For T-786 and BM-156, WES data from the PDXC were used. For the other models, WGS data from the PDX was used.
