# Supplementary material for: ATR and PKMYT1 Inhibition Resensitizes a Subset of TNBC Patient-Derived Models to Carboplatin, Inducing Mitotic Catastrophe
Source: Cancer Res Commun. 2026 May 12;6(5):1092–108. doi: 10.1158/2767-9764.CRC-25-0044 (PMC13161751; doi:10.1158/2767-9764.CRC-25-0044)
Supplement: Supplementary Figure S17 — Principal component analysis (PCA) shows a clear separation of responder and non-responder PDXs to the combination of carboplatin + ATRi. [file crc-25-0044_supplementary_figure_s17_suppsf17.pdf]

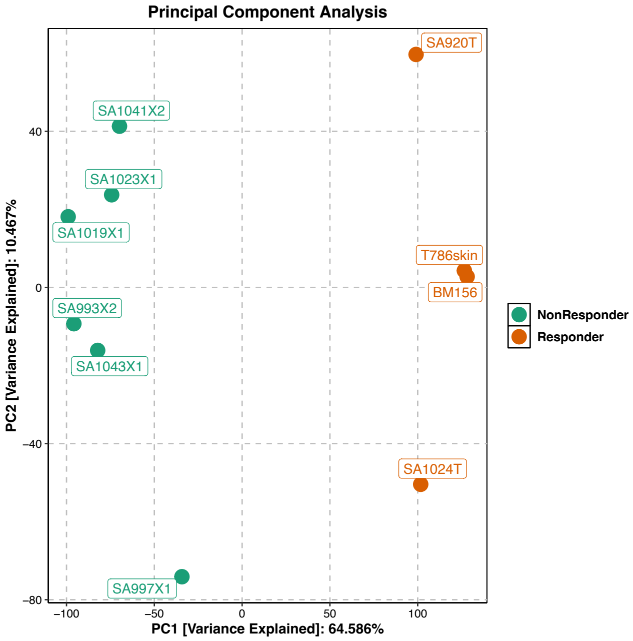

Supplementary Figure S17: Principal component analysis (PCA) shows a clear separation of responder and non-responder PDXs to the combination of carboplatin + ATRi.

The non-responders are in green SA1019X1 (PDX-1971), SA1041X2 (PDX-2076), SA1023X1 (PDX-1915), SA1043X1 (PDX-2089), SA993X2 (PDX-1924) and SA997X1 (PDX-1905)), and the responders are in orange (SA920T (PDX-1735), T-786skin (PDX T-786), BM-156 (PDX BM-156) and SA1024T (PDX-1939)).
