# Supplementary material for: ATR and PKMYT1 Inhibition Resensitizes a Subset of TNBC Patient-Derived Models to Carboplatin, Inducing Mitotic Catastrophe
Source: Cancer Res Commun. 2026 May 12;6(5):1092–108. doi: 10.1158/2767-9764.CRC-25-0044 (PMC13161751; doi:10.1158/2767-9764.CRC-25-0044)
Supplement: Supplementary Methods [file crc-25-0044_supplementary_methods_suppsm.pdf]

Supplementary methods

Combination index score

The CI is calculated by the formula[1]:

$$CI = \frac{D_1}{(D_x)_1} + \frac{D_2}{(D_x)_2}$$

Where D_1 and D_2 are drug concentrations combined with a fixed concentration of the alternate drug affecting the system at x% (in our case 50%). $(D_x)_1$ and $(D_x)_2$ stands for the concentration of the drugs alone affecting the system at x%. CI = 1 signifies an additive effect, CI < 1 synergism and CI > 1 antagonism.

PDXC generation

Briefly, tumor fragments were incubated in a mixture of Collagenase/Hyaluronidase and Dispase (STEMCELL Technologies cat#07912 and 07913) to dissociate the tissues. After centrifugation at 1100rpm and resuspension in DMEM 10% FBS, cells were filtered through a 100μM cell strainer and mouse cells were removed by using the mouse cell depletion kit from Miltenyi (Myltenyi Biotec). Human cells were then resuspended in F-medium (DMEM with 4.5g/L glucose (Wisent, #319-005-CL) with 25% Ham's F12 nutrient mixture media (gibco, #11765-054) and complemented with 7.5% FBS (Wisent, cat# 080-450), 100μg/mL penicillin-streptomycin (Wisent cat# 450-201), 1.5mM L-glutamine (Wisent cat# 609-065-EL), 0.4ug/mL hydrocortisone (Sigma-Aldrich cat# H0888), 5μg/mL insulin (Sigma-Aldrich cat# I1882), 8.4ng/mL Cholera toxin (Sigma-Aldrich cat# C8052), 10ng/ml EGF (Invitrogen cat# PHG0313),10 mM ROCK inhibitor (Y-27632 RHO/ROCK pathway inhibitor cat# 72307 (Stem Cell technologies)) and plated on lethally irradiated mouse 3T3-J2 fibroblasts (Kerafast cat#EF3003). After a few passages, co-culture with irradiated fibroblasts was interrupted and cells were grown at first in conditioned medium from irradiated fibroblasts, and subsequently, in F medium. Once in F-medium, the relative amount of mouse and human cells present in each cell line was evaluated by end-point PCR on the cell line DNA by employing Prostaglandin E Receptor 2 (PTGER2) primers[2] (**Supplementary Figure S1B and Supplementary Table S2**). When the presence of mouse DNA was detected, an additional mouse cell depletion was done.

Tumor growth inhibition measurement

Tumor growth inhibition (TGI) was measured using the formula:

$$TGI = 100 - (\frac{\Delta T}{\Delta C} * 100)$$

Where ΔT is the volume change in the treated tumors and ΔC is the volume change in the control tumors.

For mouse survival analysis Kaplan–Meier curves were performed using GraphPad (version 5.0).

Whole-exome sequencing PDXCs

DNA and RNA were extracted from PDXCs using the All Prep Qiagen kit (Qiagen) and 200ng of DNA were used for library preparation using the SureSelect XT with enzymatic fragmentation library prep kit (Agilent) according to manufacturer's instructions. Exome capture was performed using SureSelect Human All Exon v7 library prep kit (Agilent). Libraries were quantified by QuBit and BioAnalyzer DNA1000. Libraries were pooled to equimolar concentration. Sequencing was performed with the Illumina NovaSeq S4 200 cycles flowcell (2x100bp). Library preparation was made at the Institute for Research in Immunology and Cancer's Genomics Platform (IRIC) and sequencing was done at the McGill Genome Center.

Preprocessing, including removal of the molecular barcode, adapter sequences and low quality sequences using fastp. The samples were aligned to the hg37 genome using the Burrows-Wheeler Aligner bwa-mem tool. The variants were called using the GATK4 best practices workflow for somatic short variant discovery (https://gatk.broadinstitute.org/hc/en-us/articles/360035535912-Data-pre-processing-for-variant-discovery). The GATK4 best practices workflow was followed up by the FilterMutectCalls step (https://gatk.broadinstitute.org/hc/en-us/articles/360035894731-Somatic-short-variant-discovery-SNVs-Indels). Finally, variants were annotated using snpeff tool which includes functional prediction (http://pcingola.github.io/SnpEff/). Mutect2 vcf output includes a two log-odd ratios confidence score indicating the likelihood that the tumor is present. BCFtools was used to filter for only variants that passed the GATK4 tool scoring (TLOD > 6.3). In the case of the more stringent criteria, the VCF was filtered for Median mapping quality (MMQ) > 40, and allele depth (AD) > 10.

Whole genome sequencing PDXs

Whole-genome sequencing of PDX-1735, PDX-1939, PDX-1887, PDX-1915, PDX-1971, PDX-1924, PDX-1986, PDX-1991, PDX-2076 and PDX-2089 was performed and analysed as previously described[3].

RNA-sequencing PDX

Total RNA was quantified using a NanoDrop Spectrophotometer ND-1000 (NanoDrop Technologies, Inc.) and its integrity was assessed using a 2100 Bioanalyzer (Agilent Technologies). For PDX T-786 and BM-156, Ribosomal RNA were depleted from 250 ng of total RNA using Ribo-Zero rRNA Removal kit specific for HMR RNA. cDNA synthesis was achieved with the NEBNext RNA First Strand Synthesis and NEBNext Ultra Directional RNA Second Strand Synthesis Modules (New England BioLabs). The remaining steps of library preparation were done using and the NEBNext Ultra II DNA Library Prep Kit for Illumina (New England BioLabs). The libraries were normalized and pooled and the pool was loaded at 225pM on a Illumina NovaSeq S4 lane using Xp protocol as per the manufacturer's recommendations. The run was performed for 2x100 cycles (paired-end mode). Program BCL Convert 4.2.4 was then used to demultiplex samples and generate fastq reads. For the other models (PDX-1735, PDX-1939, PDX-1887, PDX-1915, PDX-1971, PDX-1924, PDX-1986, PDX-1991, PDX-2076 and PDX-2089) RNAseq results have been previously reported[3] , libraries were prepared using ssRNA-seq construction and sequenced on a HiSeq2000 according to Illumina protocols, generating 75 bp paired-end reads, as previously described[3].

RNA-sequencing bioinformatics

RNA sequencing (RNA-seq) raw reads underwent initial quality processing. Adapter sequences were trimmed and low-quality reads filtered using CutAdapt[4] via Trim Galore[5]. Quality control metrics for raw and processed reads were assessed using FastQC[6] and SAMtools[7] respectively. Trimmed reads were aligned to the human reference genome (GRCh38.p13) using the STAR aligner[8], guided by Gencode Human Release 38 (v38) annotations in GTF format[9] after a disambiguation process, in which reads that preferentially map to the human genome are isolated from those mapping to the mouse genome, ensuring that only the human-mapping reads are retained for PDX and PDXC samples. Post-alignment quality was

summarized using MultiQC[10]. Gene-level expression quantification was performed with featureCounts[11] to count reads mapped to each gene. Transcript per million (TPM) values were computed using Salmon[12], both utilizing Gencode v38 annotations. Differential expression analysis between conditions was conducted using DESeq2[13], preceded by batch effect correction with the ComBat tool from the SVA package[14] for PDX samples. This analysis accounted for biological variability and controlled the false discovery rate (FDR). Genes with low counts (fewer than 10 reads in more than half of the samples) were excluded from further analysis. Genes were considered significantly dysregulated if they met an FDR-adjusted p-value cutoff of 0.05 and a fold change cutoff of 2. Gene Set Enrichment Analysis (GSEA) was performed on all expressed genes, ranked by decreasing log2 fold change, to identify biologically enriched pathways. Enrichment analysis utilized the fgsea package[15] in R, referencing publicly available gene sets from MSigDB[16], including C2 (KEGG Legacy), C5 (Gene Ontology Biological Process), and HALLMARK signatures. Enrichment results were further analyzed and visualized using the clusterProfiler[17] and ggplot2[18] R packages.

References

1. Chou, T.C. & Talalay, P. Quantitative analysis of dose-effect relationships: the combined effects of multiple drugs or enzyme inhibitors. *Adv Enzyme Regul* **22**, 27-55 (1984).
2. Alcoser, S.Y., *et al.* Real-time PCR-based assay to quantify the relative amount of human and mouse tissue present in tumor xenografts. *BMC Biotechnol* **11**, 124 (2011).
3. Savage, P., *et al.* Chemogenomic profiling of breast cancer patient-derived xenografts reveals targetable vulnerabilities for difficult-to-treat tumors. *Commun Biol* **3**, 310 (2020).
4. Martin, M. Cutadapt Removes Adapter Sequences From High-Throughput Sequencing Reads. *EMBnet* **17**(2011).
5. Krueger, F. Krueger F. 2015. Trim Galore: a wrapper tool around Cutadapt and FastQC to consistently apply quality and adapter trimming to FastQ files. *Babraham Institute* (2015).
6. Andrew, S. FastQC: A Quality Control Tool for High Throughput Sequence Data. *Babraham Institute* (2010).
7. Heng Li, B.H., Alec Wysoker, Tim Fennell, Jue Ruan, Nils Homer, Gabor Marth, Goncalo Abecasis, Richard Durbin; 1000 Genome Project Data Processing Subgroup. The Sequence Alignment/Map format and SAMtools. *Bioinformatics* **25**, 2078–2079 (2009).
8. Dobin, A., *et al.* STAR: ultrafast universal RNA-seq aligner. *Bioinformatics* **29**, 15-21 (2013).
9. Frankish, A., *et al.* GENCODE reference annotation for the human and mouse genomes. *Nucleic Acids Res* **47**, D766-D773 (2019).
10. Philip Ewels, M.M., Sverker Lundin, Max Käller. MultiQC: summarize analysis results for multiple tools and samples in a single report. *Bioinformatics* **32**, 3047–3048 (2016).
11. Liao, Y., Smyth, G.K. & Shi, W. featureCounts: an efficient general purpose program for assigning sequence reads to genomic features. *Bioinformatics* **30**, 923-930 (2014).
12. Patro, R., Duggal, G., Love, M.I., Irizarry, R.A. & Kingsford, C. Salmon provides fast and bias-aware quantification of transcript expression. *Nat Methods* **14**, 417-419 (2017).
13. Michael I Love, W.H.S.A. Moderated estimation of fold change and dispersion for RNA-seq data with DESeq2. *Genome biology* **15**(2014).
14. Jeffrey T. Leek, W.E.J., Hilary S. Parker, Andrew E. Jaffe, and John D. Storey. The sva package for removing batch effects and other unwanted variation in high-throughput experiments. *Bioinformatics* **28**, 882-883 (2012).
15. Gennady Korotkevich, V.S., Nikolay Budin, Boris Shpak, Maxim N. Artyomov, Alexey Sergushichev. Fast gene set enrichment analysis. *BioRxiv* (2021).
16. Subramanian, A., *et al.* Gene set enrichment analysis: a knowledge-based approach for interpreting genome-wide expression profiles. *Proc Natl Acad Sci U S A* **102**, 15545-15550 (2005).
17. Wu, T., *et al.* clusterProfiler 4.0: A universal enrichment tool for interpreting omics data. *Innovation (Camb)* **2**, 100141 (2021).
18. H, W. ggplot2: Elegant Graphics for Data Analysis. *Springer-Verlag New York* (2016).
